# Genetic Architecture and Candidate Genes for Deep-Sowing Tolerance in Rice Revealed by Non-syn GWAS

**DOI:** 10.3389/fpls.2018.00332

**Published:** 2018-03-16

**Authors:** Yan Zhao, Weipeng Zhao, Conghui Jiang, Xiaoning Wang, Huaiyang Xiong, Elena G. Todorovska, Zhigang Yin, Yanfa Chen, Xin Wang, Jianyin Xie, Yinghua Pan, Muhammad A. R. Rashid, Hongliang Zhang, Jinjie Li, Zichao Li

**Affiliations:** ^1^Key Lab of Crop Heterosis and Utilization of Ministry of Education and Beijing Key Lab of Crop Genetic Improvement, China Agricultural University, Beijing, China; ^2^Institute of Food Crops, Hainan Academy of Agricultural Sciences, Haikou, China; ^3^AgroBioInstitute, Bulgarian Agricultural Academy, Sofia, Bulgaria; ^4^Rice Research Institute, Guangxi Academy of Agricultural Sciences, Nanning, China; ^5^Plant Breeding and Genetics Lab, University of Agriculture Faisalabad, Vehari, Pakistan

**Keywords:** deep-sowing tolerance, genome-wide association study, mesocotyl length, non-synonymous SNP, *Oryza sativa*

## Abstract

Dry direct-seeding of rice is rapidly increasing in China, but variable planting depth associated with machine sowing can lead to low seedling emergence rates. Phenotype analysis of 621 rice accessions showed that mesocotyl length (ML) was induced by deep soil covering and was important in deep-sowing tolerance in the field. Here, we performed and compared GWAS using three types of SNPs (non-synonymous SNP, non-synonymous SNPs and SNPs within promoters and 3 million randomly selected SNPs from the entire set of SNPs) and found that Non-Syn GWAS (GWAS using non-synonyomous SNP) decreased computation time and eliminated confounding by other loci relative to GWAS using randomly selected SNPs. Thirteen QTLs were finally detected, and two new major-effect genes, named *OsML1* and *OsML2*, were identified by an integrated analysis. There were 2 and 7 non-synonymous SNPs in *OsML1* and *OsML2*, respectively, from which 3 and 4 haplotypes were detected in cultivated rice. Combinations of superior haplotypes of *OsML1* and *OsML2* increased ML by up to 4 cm, representing high emergence rate (85%) in the field with 10 cm of soil cover. The studies provide key loci and naturally occurring alleles of ML that can be used in improving tolerance to dry direct-seeding.

## Introduction

Rice is one of the most important food crops, feeding more than one-half of the world population. Rice planting is currently carried out in two ways, namely transplanting and direct-seeding (Farooq et al., 2011). Transplanting is a traditional system based on transplanting of seedlings from seedbeds to paddy fields, and is used to ensure high seedling emergence and uniform plant density (Farooq et al., 2011; Wu et al., 2015). Transplanting by hand remains the predominant method in China, accounting for more than 90% of the total area. Direct-seeding is the major cultivation method in Europe and the United States due to mechanized farming practices, and savings in labor and time (Farooq et al., 2011). Recently, dry direct-seeding is increasing rapidly in China, but variable planting depths associated with machine sowing cause agronomic problems that prevent the achievement of optimum plant populations (Farooq et al., 2006a,b). Poor emergence and weak seedling establishment caused by deep soil greatly restrict the application of direct-seedling technology. Thus, an understanding of the genetic mechanisms affecting the plant establishment by dry direct-seeding is needed to support breeding solutions.

The mesocotyl is an embryonic structure between the scutellar node and coleoptilar node, and it can directly push the shoot tip above the soil surface during germination (Lee H. S. et al., 2012). Thus, mesocotyl length (ML) is an important trait affecting plant establishment under deep sown conditions in rice (Turner et al., 1982; Wu et al., 2015; Lu et al., 2016). Earlier studies showed that drill-seeded semi-dwarf rice genotypes emerge more slowly and less uniformly than non-dwarf types with long mesocotyls (Turner et al., 1982). However, further study indicated there was no correlation between ML and mature plant traits such as plant height and internode length (Mgonja et al., 1993). Thus, it should be possible to breed semi-dwarf accessions with long mesocotyls and to identify genes determining ML. Mesocotyls can elongate in dark and water-covered situations as well as under deep sown conditions (Feng et al., 2017). Under dark and water-covered conditions, elite germplasm with long mesocotyls were identified in different subpopulations and ecotypes (Redoña and Mackill, 1996a; Wu et al., 2005; Luo et al., 2007). The previous studies generally showed that accessions with long mesocotyls were rare in cultivated rice. Some elite *indica* accessions with long mesocotyls were identified under direct-seeding conditions of 5 cm depth of sand cover (Lu et al., 2016). Given that mesocotyl elongation in soil-sand culture differs from elongation under dark and water-covered conditions (Simon et al., 2011), further deep-sowing field experiments were needed to identify germplasm with long mesocotyls and to determine the relationship between ML and emergence rate (ER).

Traditional bi-parental linkage mapping has been fully applied to detect QTL for complex traits, including ML. Five QTLs for ML were first identified using an F_2_ population of 204 plants from a cross between a low-vigor *japonica* “Labelle” and a high-vigor *indica* “Black Gora” (Redoña and Mackill, 1996b). Using a DH population crossed between *indica* and *japonica*, eight QTLs for ML were detected (Cao et al., 2002). Additionally, 11, 8, and 27 QTLs for ML were identified by using RIL population, respectively (Cai and Morishima, 2002; Ouyang et al., 2005; Huang C. et al., 2010). In a subsequent study, linkage mapping of ML was conducted by using BIL population from a cross between the cultivated rice and weedy rice with long ML (Lee H. et al., 2012). These identified genomic regions that were associated with ML, and were conducive to breeding for deep-sowing tolerance and gene cloning.

Previous studies demonstrated that genes *OsBRI1, D10, D17, D27, D3, D14, PTOX1*, and *OsTCP5* were involved in ML by comparison of mutants and wild type (Hu et al., 2010, 2014; Gao et al., 2014; Tamiru et al., 2014; Kameoka and Kyozuka, 2015). Studies showed that the elongation of mesocotyls were regulated by strigolactones and cytokinins during germination of rice seeds in darkness (Hu et al., 2010, 2014; Chen et al., 2014; Tamiru et al., 2014; Kameoka and Kyozuka, 2015). Other reports indicated that brassinosteroids, ethephon and gibberellic acid were also involved (Watanabe et al., 2015; Liang et al., 2016). Dynamic transcriptome analysis suggested plant hormone signal transduction, α-linolenic acid metabolism and diterpenoid biosynthesis were critical processes of mesocotyl growth that were inhibited by light (Feng et al., 2017). Importantly, a single natural variation of the *GY1* gene for ML was identified from 3,000 accessions, and was investigated by map-based cloning (Xiong et al., 2017). The gene functioned at the initial step of jasmonic acid biosynthesis to repress mesocotyl and coleoptile elongation. Despite these reports, identification of natural variation in ML may be a better way to find genes associated with ML for breeding and for gaining insights into the molecular basis of variation in ML.

The advent of the next-generation sequencing technology offers abundant genetic information and a solid basis for genome-wide association studies (GWAS). Compared with conventional linkage mapping, GWAS explores a wider range of natural variation and enables a greater number of significant SNPs to be identified. By GWAS of 1,019,883 SNPs in 270 rice accessions, 13 SNPs were identified to be highly associated with ML of rice plants grown in water (Wu et al., 2015). Another GWAS of 4,136 SNPs in 469 *indica* rice accessions identified 17 loci for ML, explaining 19.31% of the phenotypic variation (Lu et al., 2016). These two preliminary GWAS provided reliable QTL regions for ML, although it was hard to distinguish functional loci. High density sequencing and GWAS of a large representative collection of germplasm was necessary to gain further insights into loci and naturally occurring alleles for ML.

To breed rice accessions for dry direct-seeding, the following problems needed to be solved: (1) identification of germplasm with long mesocotyls and investigation of the relationship between ML and seedling ER under deep-sowing conditions in the field; and (2) exploration of QTLs or candidate genes for ML that can be further used in molecular breeding by marker assisted selection. In this study, we evaluated ML and ER of a large population of germplasm under deep sown conditions in the field and identified several accessions with long mesocotyls as possible breeding parents. More than 15 million SNPs were identified in the germplasm following sequencing at an average depth of 15X. We first screened for non-synonymous SNPs linked to ML and performed GWAS between ML and non-synonymous SNPs. Candidate genes/loci for ML were verified by an integrated analysis of GWAS, linkage mapping, allelic frequency differences between phenotypic pools, expression, and sequence alignment.

## Materials and methods

### Materials and sequencing

A total of 621 cultivated rice accessions from the 3000 Rice Genome Project (3KRGP) (Li J. Y. et al., 2014; Alexandrov et al., 2015) formed the materials for identification of ML QTLs. The collection was based on broadly genetic diversity, and comprised mini-core collections selected from an original core set of 4,310 primary accessions of Chinese cultivated rice (Zhang et al., 2011), and 402 lines in the International Rice Molecular Breeding Network (Yu et al., 2003). The sequencing data of the 621 accessions were directly from the 3KRGP, which have an average sequencing depth of 15X and generated >15 million SNPs when compared with the Nipponbare reference genome (Li Z. et al., 2014).

### Phenotyping

Based on previous studies (Turner et al., 1982; Lu et al., 2016), we chose 10 cm depth of soil cover to measure MLs of different accessions, which could exceed the maximum capacity of mesocotyl elongation in rice. We then designed field experiments with 10 cm depth of soil cover at the Experiment Station of China Agricultural University, Beijing. Ten full and uniformed seeds of each accession were planted in the field. A 10 cm soil layer was placed over the seeds followed by sprinkling with adequate water. The field experiment was conducted in summer of 2014 in two fields as two replications (Figure S1). After 10 days, MLs of all seedlings were measured with a ruler.

For an experiment in plastic boxes, three groups of five accessions with ML in the range of 0–0.5, 2–3, and 5–6 cm were planted on a thick soil layer (Figure S2). Each accession was planted in one column with 30 plants as repetition. The seeds in separate boxes were covered with 1, 3, 5, and 7 cm of soil followed by sprinkling with adequate water. MLs of all seedlings were measured after 10 days.

### Non-synonymous SNPs and population genetic analysis

Based on information on coding sequence (CDS) coordinates and transcript from MSU-RGAP 7, we separated the non-synonymous SNPs from a total of 15 million SNPs using an in-house Perl script. A neighbor-joining (N-J) tree was generated from more than 75,000 randomly selected SNPs using Tassel 5 and Mega 6 software. Principal components (PC) and kinship matrix were calculated by software GAPIT to verify population structure using more than 3 million SNPs with minor allele frequency >0.05 and missing rates <0.5. LD heatmaps of two important QTLs identified in GWAS were generated using the R package “LD heatmaps.” Candidate regions were identified using an *r*^2^ > 0.6. Nucleotide diversity (π) (Nei, 1987) and Tajima's D (Tajima, 1989) were calculated using an in-house Perl script.

### Screening of SDSs and ESDSs for mesocotyl length

For screening of the SDSs (SNPs with significant differences in allele frequency between polar pools, *p* < 0.05) and ESDSs (SNPs with extremely significant differences in allele frequency between polar pools, *p* < 0.01) associated with ML by bulked segregant analysis, we selected accessions with polar ML from typical *indica* and *japonica* accessions, followed by chi-squared tests of allele frequency for each non-synonymous SNP. To identify the SDS- and ESDS-enriched windows, we performed a permutation test to obtain significant thresholds by random shuffling of 10,000 iterations of SNP numbers of all 500 kb sliding windows along the entire genome. We finally set 99th percentiles of SDS and ESDS numbers of permutation tests as threshold values.

### Comparison of GWAS using three types of SNPs for mesocotyl length

To perform GWAS efficiently it is important to eliminate false positives from the population structure and to identify family relationships in natural population. The first three PCs were used to construct the PC matrix. We performed GWAS using CMLM with PC and kinship, which accounts for population structure and identifies the optimal group kinship matrix (Zhang et al., 2010a). Previous studies indicated that a trait-specific kinship derived from weighted SNPs has better genomic prediction accuracy than kinship derived from all SNPs (Zhang et al., 2010b; Wang et al., 2014). Thus, we performed and compared GWAS of the CMLM using 3 groups of SNPs to search for target genes for ML. Group I included non-synonymous SNPs; group II included non-synonymous SNPs and SNPs located in 5′ flanking sequences of genes (≤1 Kb upstream of the first ATG); and group III included 3 million randomly selected SNPs from the entire SNP set.

Given that the default parameters were too strict for detecting significant associations when the threshold was derived from the total number of markers, we used the formula “−log_10_(0.01/effective number of SNPs with a *p*-value less than 0.01),” i.e., the threshold at a significance level of 1% after Bonferroni-adjusted multiple test correction (Pan et al., 2015). False discovery rate (FDR) was performed to compare with the threshold value (Benjamini and Hochberg, 1995). All signals at a significance level of 0.01 after Bonferroni-adjusted multiple test corrections were up to a significance level of FDR (*p* < 0.05).

### RNA extraction and qRT-PCR

Total RNA was extracted from mesocotyls of 6 rice accessions using RNAiso Plus (Takara). The cDNA was generated in 25 μl reaction mixtures containing 2 μg Dnase I-treated RNA, 200 U M-MLV reverse transcriptase (Takara), 40 U Recombinant RNase Inhibitor (Takara) and 0.1 μM oligo (dT)_18_ primer. qRT-PCR was performed in total volumes of 10 μl containing 5 μl SYBR premix EX Taq (Takara), 0.2 μl Rox Reference Dye II (Takara), 0.4 mM gene-specific primers and 0.5 ul of cDNA on an ABI 7500 Real time PCR system (Applied Bio-systems). The gene LOC_Os03g50885 was used as an internal reference.

## Results

### Population characterization and phenotypic variation in mesocotyl length

**Figure 1:**
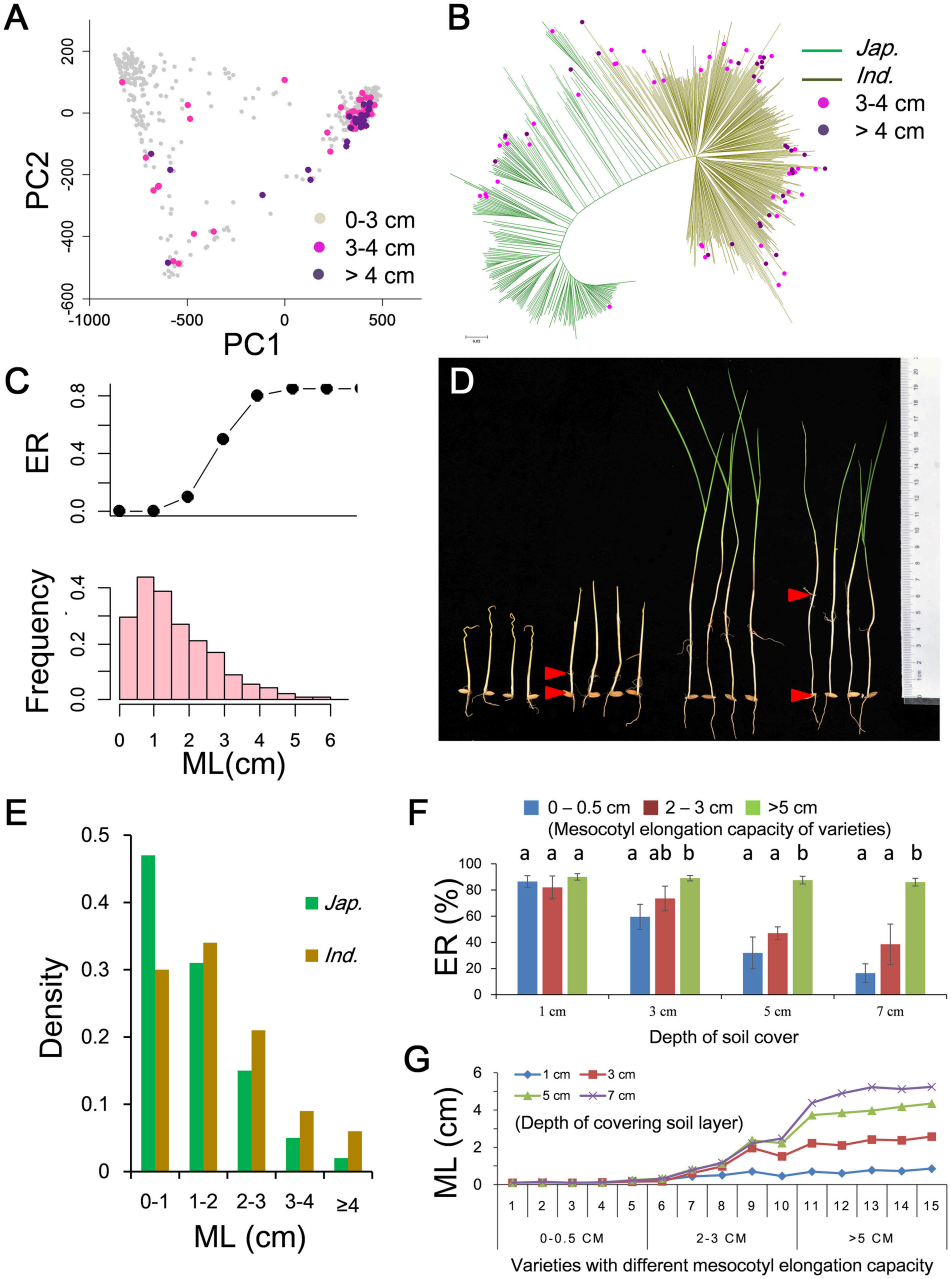
Mesocotyl lengths (MLs) in 621 cultivated rice and relationship among ML, emergence rate (ER) and planting depth. **(A)** Principal component plot and distribution of accessions with different ML. **(B)** Neighbor-joining tree and distribution of accessions with ML of more than 3 cm on the tree. **(C)** Relationship between ER and ML (upper) and histogram of ML (lower) in field plantings with 10 cm of soil cover. **(D)** Differentiation of MLs in cultivated accessions. **(E)** Comparison of MLs of *indica* and *japonica*. **(F)** Relationship between ER and ML in box plantings with different depths of soil cover. Different letters above bars indicate significant differences (*p* < 0.05) detected by Duncan's multiple range test. **(G)** Relationship between ML and depth of soil cover for accessions with different mesocotyl elongation capacities.

The PC analysis showed that PC1 could explain more than 90% genetic variation, suggesting the 621 accessions could be classified in two major subpopulations (Figure 1A and Figure S3). The result was also supported by the N-J tree and kinship plot (Figure 1B and Figure S3). Therefore, referring to the reported classification information of the sample (Yu et al., 2003; Zhang et al., 2011), we divided the population into two subgroups, including 390 *indica* and 231 *japonica* accessions. Large variations in ML and ER were observed in repeated phenotypic assays in the field with 10 cm of soil cover, with ML ranging from 0.1 to 6.19 cm and ER ranging from 0 to 85% (Figures 1C,D and Table S1). Generally, variation in ML in rice fitted a negative binomial distribution, and most of accessions had short mesocotyls ranging from 0 to 1 cm (Figure 1C). There was high ER (85%) when ML exceeded 4 cm in the field with 10 cm of soil cover (Figure 1C and Figure S4). Whereas most accessions had short ML there were significant differences in ML between *indica* and *japonica* (Table S2). There was a higher proportion of accessions with ML of 0–1 cm in *japonica* (46.7%) than in *indica* (30.2%) (Figure 1E). However, more *indica* accessions had long mesocotyls of 4 cm or more than in *japonica* (Figure 1E). Finally, we identified 23 *indica* and 4 *japonica* accessions having deep-sowing tolerance with ER of about 80% and with ML of more than 4 cm (Figures 1A,B and Table S3). The average plant height of these accessions was about 104 cm. Considering the weak correlations between ML and plant height in rice (Table S4), these accessions were considered to be potentially useful parental germplasm for breeding.

To gain further insight into the relationship among ML, ER and depth of soil cover we measured the ER of accessions with different ML at different depths of soil cover (Figure S2). Three groups of five accessions were selected according to ML in the range of 0–0.5, 2–3, and 5–6 cm in the field. The 15 accessions were planted on a thick soil layer in plastic boxes. The seeds in separate boxes were covered with 1, 3, 5, and 7 cm of soil. Under shallow sowing conditions (covered with 1 and 3 cm of soil), the three groups accessions had higher ER of 60–90% (Figure 1F) and shorter ML of 0.1–2.6 cm (Figure 1G). There were no significant correlations between ML and ER in boxes covered with 1 or 3 cm of soil. Under deep sowing (covered with 5 and 7 cm of soil), only five accessions with long ML (>5 cm) had higher ER (≈87%) (Figure 1F), and the other two groups with ML in the range of 0–0.5 and 2–3 cm showed lower ER of 16 and 39%, respectively (Figure 1F). There were highly significant correlations between ML and ER with Pearson correlation coefficients of 0.81 and 0.78 in boxes covered with 5 and 7 cm of soil, respectively. The results showed that ML was induced by the depth of soil cover, and greater ML led to improved ER under deep sowing conditions.

### Characteristics of non-synonymous SNPs along the entire genome and screening of SDSs and ESDSs

**Figure 2:**
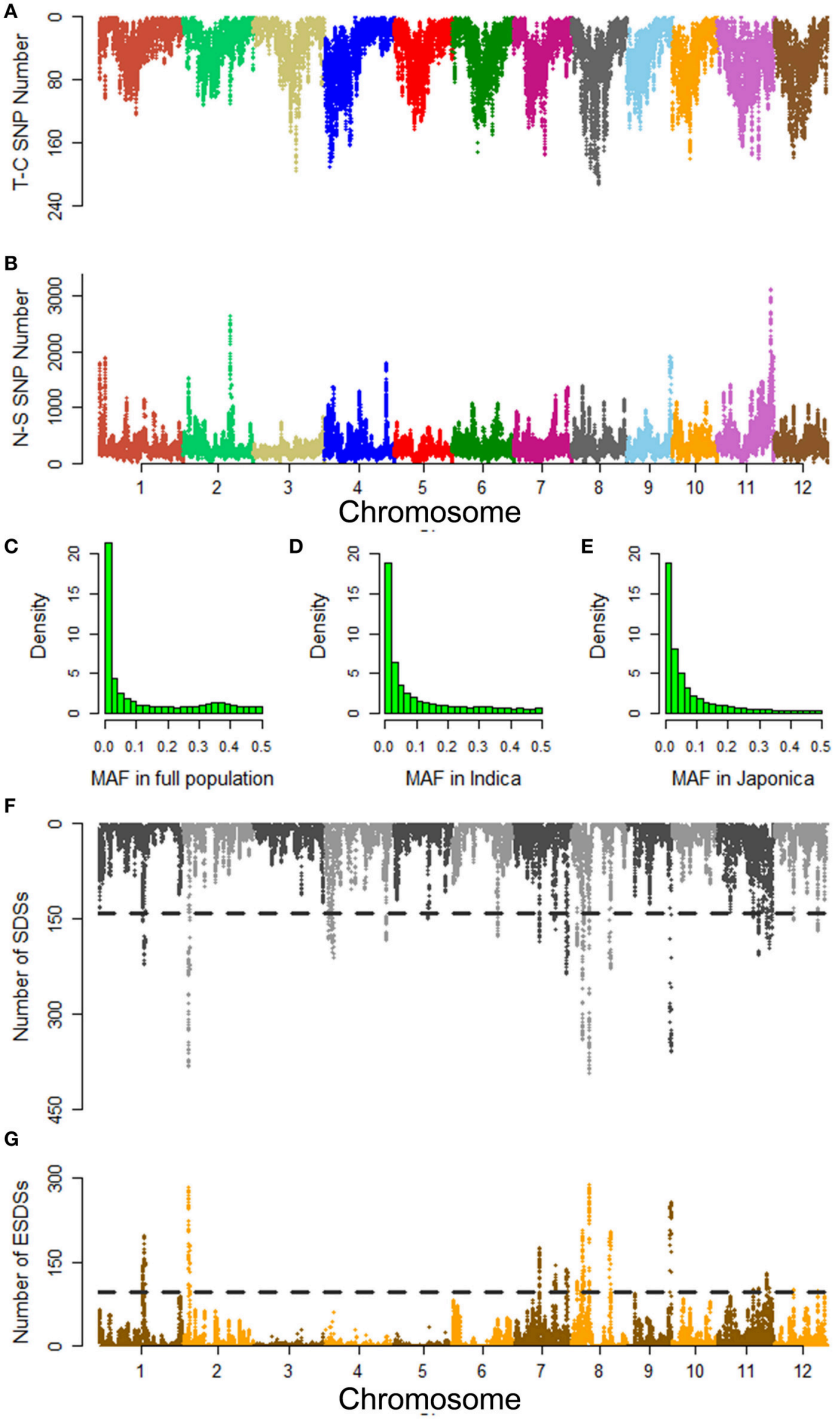
Summary of non-synonymous SNPs detected in the full population and their distribution associated with mesocotyl length in the *indica* genome. **(A)** Termination codon SNP (T-C SNP) along the genome. **(B)** Non-synonymous SNPs (N-S SNP) along the genome. Histograms of minor allele frequencies (MAFs) of non-synonymous SNPs in the **(C)** full population, **(D)** *indica*, and **(E)** *japonica*. Distributions of **(F)** SDSs and **(G)** ESDSs along the *indica* genome. SDS and ESDS show SNPs with significant and extremely significant differences (*p* < 0.05; *p* < 0.01) in allele frequency between polar pools. For each 500 kb sliding window, the numbers of SDSs and ESDSs were plotted on the entire genome. The sliding step is 50 kb. Adjacent chromosomes are delineated using different colors. Horizontal black lines show the thresholds for the 99th percentile of 10,000 permutations of the SDS and ESDS numbers.

Polymorphisms causing protein-coding differences are most likely to be important functional loci associated with agronomic traits (Yano et al., 2016). Here, we focused on non-synonymous SNPs in all 50,086 annotated genes from MSU-RGAP 7 except transposons and retrotransposons. The 572,511 non-synonymous SNPs were screened from 15 million SNPs across the whole genomes of all accessions. Among them 73,000 SNPs were found to cause premature terminations (Figures 2A,B). The analysis of minor allele frequencies (MAFs) indicated that 54, 55, and 60% of the non-synonymous SNPs could be considered as rare variants in the full population, and *indica* and *japonica* for groups with low MAF (<0.05) (Figures 2C–E).

To explore non-synonymous SNPs associated to ML, we constructed separate pools with extreme differences in ML based on conventional bulked segregant analysis for *indica* and *japonica*. To reduce genetic differences unrelated to ML between polar pools, we took no account of accessions at PC1 values ranging from −400 to 300. The accessions with PC1 <−400 were defined as typical *indica*, and those with PC1 >300 were considered to be typical *japonica*. Each pool included 20 accessions of each subgroup (Table S5). On the basis of chi-squared tests of allele frequency between the polar pools, we selected out the SDSs and ESDSs associated with ML (Figures 2F,G and Figure S5). As a result we obtained 35,911, and 40,615 SDSs in *indica* and *japonica*, respectively. Among them, there were 19,073 and 26,055 ESDSs associated with ML in each subgroup. These SNPs appeared to be randomly distributed over the entire genome except for a few enriched peaks. Using thresholds of extreme significance for each window established by permutation tests we selected the regions with peaks higher than the threshold as candidate loci associated with ML (Figures 2F,G and Figure S5).

### Comparison of GWAS using three types of SNPs for mesocotyl length

**Figure 3:**
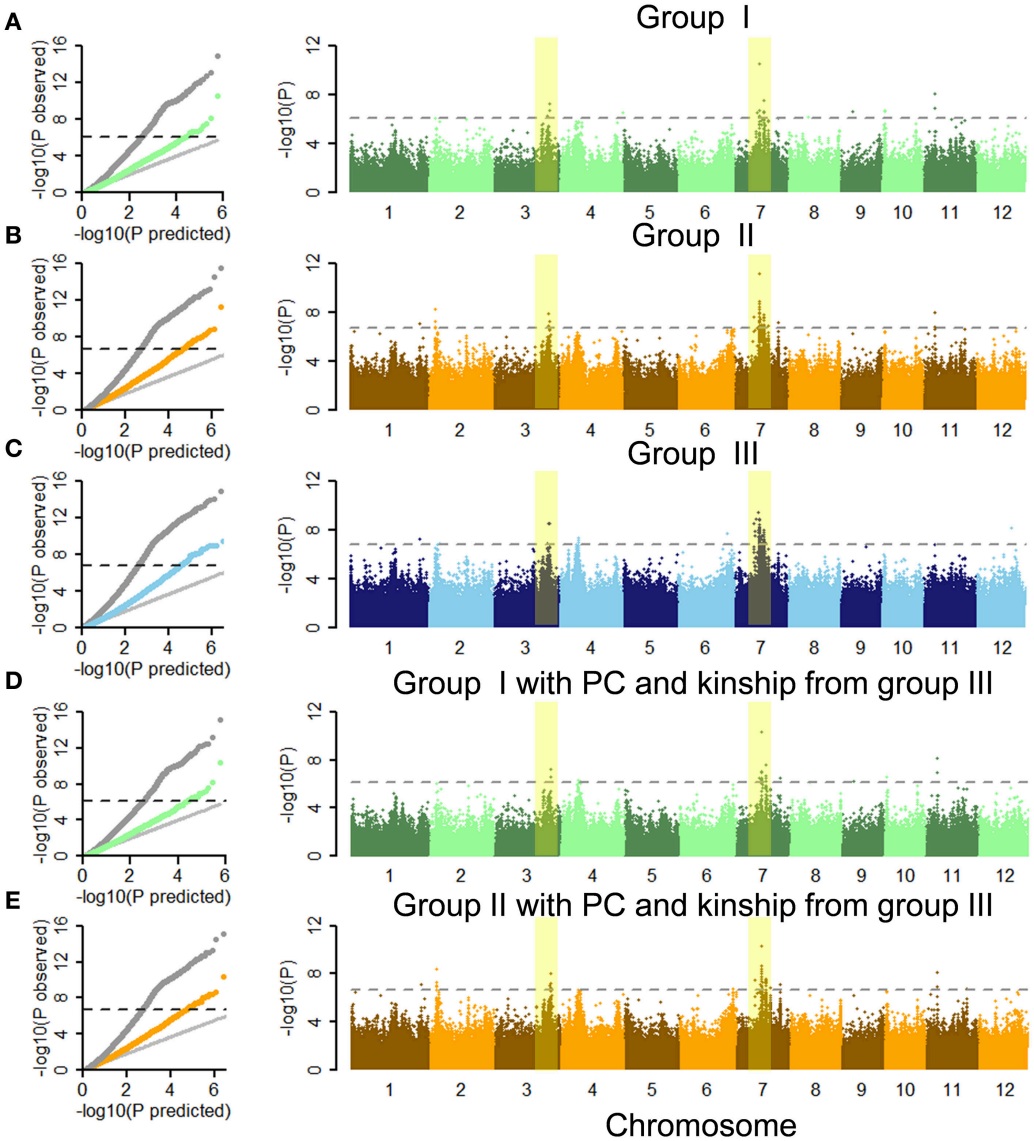
Genome-wide association studies of mesocotyl length under CMLM in full population using three sets of SNPs and different PC and kinship. Quantile-quantile plots and Manhattan plots of the CMLM using groups **(A)** I, **(B)** II, and **(C)** III in full population. Quantile-quantile plots and Manhattan plots of CMLM using groups **(D)** I and **(E)** II with PC and kinship derived from group III in full population. In quantile-quantile plots, gray dots show GLM, and other colored points show CMLM. The horizontal black dashed lines in Manhattan plots of CMLM show thresholds at *p* = 0.01 after Bonferroni-adjusted multiple test correction. Yellow stripes show two important signals in three GWAS using three sets of SNPs in the full population.

Quantile-quantile (Q-Q) plots for ML showed that CMLM accounted for false positives arising from the population structure and family relationships in our population on the basis of GLM (Figures 3A–C). By performing GWAS using each group separately, we identified 23, 53, and 91 SNPs for ML at significance levels of 0.01 after Bonferroni-adjusted multiple test correction in the full population (Figures 3A–C and Table 1 and Tables S6, S7). Due to lower genome-wide linkage disequilibrium (LD) decay rates in *indica* and *japonica* at 123 and 167 kb (Huang X. et al., 2010), adjacent significant SNP with distances <170 kb were merged into an independent QTL. Thirteen, 11 and 20 QTLs were identified by GWAS using the separate groups (Table 1 and Tables S6, S7). We also performed GWAS of ML in *indica* with its wider phenotypic variation (Figure S6). Here we identified 3, 5, and 4 QTLs, including 11, 21, and 17 SNPs associated with ML, respectively (Tables S8–S10). With GLM, these SNPs and QTLs showed higher −log(*p*) values as well (Figures S7, S8 and Table 1 and Tables S6–S10). By comparison of GWAS results using the three sets of SNPs, five QTL in the full population (named as *qFML3-2, qFML7-2, qFML7-3, qFML7-4*, and *qFML11-1*) from GWAS using group I were detected in GWAS using groups II and III; one QTL in *indica* (named as *qIML7-2*) from GWAS using group I were also detected in GWAS of groups II and III. The results demonstrated that Non-Syn GWAS (GWAS using SNPs from group I) was a feasible method to identify QTL regions as well as GWAS of 3 million randomly selected SNPs.

**Table 1:** Summary of SNPs associated with ML by GWAS using CMLM and group I in the full population.

**QTL**	**Gene**	**Position**	**−log(*p*)^a^**	**−log(*p*)^b^**	**−log(*p*)^c^**	***x*^2^-value^d^**	***x*^2^-value^e^**	**SNP variation**	**Amino acid variation**	**MAF**	**Functional annotation**
*qFML2-1*	LOC_Os02g07480	Chr2_3858289	6.12	8.13	6.07	1.39	0	G/T	T/N	0.011	Transglycosylase SLT domain containing protein, expressed
*qFML3-1*	LOC_Os03g51340	Chr3_29376315	6.26	9.65	4.66	11.86	0.51	C/T	P/L	0.074	Expressed protein
*qFML3-2*	LOC_Os03g53320	Chr3_30603087	7.31	12.69	7.24	20.72	0.51	G/A	A/V	0.069	Hypothetical protein
	LOC_Os03g53340	Chr3_30606285	6.74	11.89	6.6	19.12	0.51	G/T	D/E	0.076	HSF-type DNA-binding domain containing protein, expressed
*qFML4-1*	LOC_Os04g58590	Chr4_34837207	6.53	3.52	3.54	0	2.37	A/T	L/P	0.01	RNA recognition motif containing protein, putative, expressed
*qFML7-1*	LOC_Os07g22360	Chr7_12552125	6.53	5.78	6.54	9.26	0	G/A	V/I	0.047	Expressed protein
*qFML7-2*	LOC_Os07g23990	Chr7_13602658	6.77	9.71	7.04	26.52	0.51	A/T	M/L	0.366	Tetratricopeptide repeat domain containing protein, putative, expressed
	LOC_Os07g24010	Chr7_13611491	10.51	14.9	10.35	31	0	A/T	S/T	0.26	Hypothetical protein
	LOC_Os07g24170	Chr7_13728692	6.73	9.66	6.95	28.82	0.51	T/A	N/K	0.39	Expressed protein
		Chr7_13728704	6.27	9.1	6.43	26.45	0.51	G/T	Q/H	0.388	
		Chr7_13729329	6.18	9.26	6.32	31.25	0	G/A	V/M	0.393	
	LOC_Os07g24190	Chr7_13746039	6.32	9.05	6.53	24.22	0	C/T	M/I	0.385	CESA3 - cellulose synthase, expressed
*qFML7-3*	LOC_Os07g25460	Chr7_14579544	6.11	9.06	5.96	22.65	0.51	G/A	R/C	0.394	Ankyrin repeat domain containing protein, expressed
*qFML7-4*	LOC_Os07g27610	Chr7_16129890	7.58	13.11	7.56	33.63	0	G/A	R/Q	0.438	Expressed protein
	LOC_Os07g27630	Chr7_16135146	6.57	12	6.5	29.53	0	G/A	S/L	0.185	Expressed protein
	LOC_Os07g27680	Chr7_16151805	6.65	12.31	6.65	33.63	0	T/C	T/A	0.457	Expressed protein
*qFML7-5*	LOC_Os07g39660	Chr7_23772528	6.14	9.65	6.47	27.42	0	A/T	H/L	0.221	Hypothetical protein
*qFML8-1*	LOC_Os08g17350	Chr8_10613231	6.17	6.24	6.18	0	0	G/A	S/F	0.004	Expressed protein
*qFML9-1*	LOC_Os09g11800	Chr9_6598055	6.63	8.52	6.25	2.37	0	T/A	V/E	0.009	Expressed protein
*qFML10-1*	LOC_Os10g03730	Chr10_1681425	6.64	9.13	6.63	4.8	0	T/C	E/G	0.024	OsFBX347 - F-box domain containing protein, expressed
	LOC_Os10g03780	Chr10_1713500	6.69	9.5	6.59	5.64	0	G/A	Q/^*^	0.02	OsFBX351 - F-box domain containing protein, expressed
*qFML11-1*	LOC_Os11g10920	Chr11_6031396	8.07	9.64	8.18	2.37	0	A/C	V/G	0.011	Carboxyl-terminal proteinase, putative, expressed
	LOC_Os11g10990	Chr11_6065939	6.91	8.42	6.92	2.37	0	C/T	G/R	0.01	Heat shock protein DnaJ, putative, expressed

a *−log(p) are association signals of CMLM using PC and kinship derived from non-synonymous SNPs (group I)*.

b *−log(p) are association signals of GLM using PC derived from group I*.

c *−log(p) are association signals of CMLM using PC and kinship derived from group III*.

d *x^2^-value are chi-squared tests between polar pools in indica*.

e *x^2^-value are chi-squared tests between polar pools in japonica*.

Due to PC and kinship derived from genetic markers different sets of markers result in different PC and kinship. That is the biggest difference among GWAS using three groups of SNPs. To assess the effect of the original GWAS using groups I and II, we performed complementary GWAS between ML and SNPs in these groups using PC and kinship data derived from group III. As shown by the Q-Q and Manhattan plots, similar GWAS results were obtained using PC and kinships derived from groups I, II, and III in CMLM (Figure 3 and Figures S6, S9). By further comparing peak −log(*p*) values of each QTL between the original and complementary GWAS using the full population 9 of 13 and 8 of 11 QTLs in the original GWAS of the full population had higher signals than in the complementary GWAS using groups I and II, respectively (Table 1 and Table S6). Furthermore, all 3 and 5 QTLs in the original GWAS in *indica* also had higher signals than the complementary GWAS using groups I and II (Tables S8, S9). These results indicated that PC and kinship derived from groups I and II effectively reduced the frequency of false positives. Moreover, Non-Syn GWAS markedly reduced the computational burden.

### Exploration of candidate gene *OsML1*

**Figure 4:**
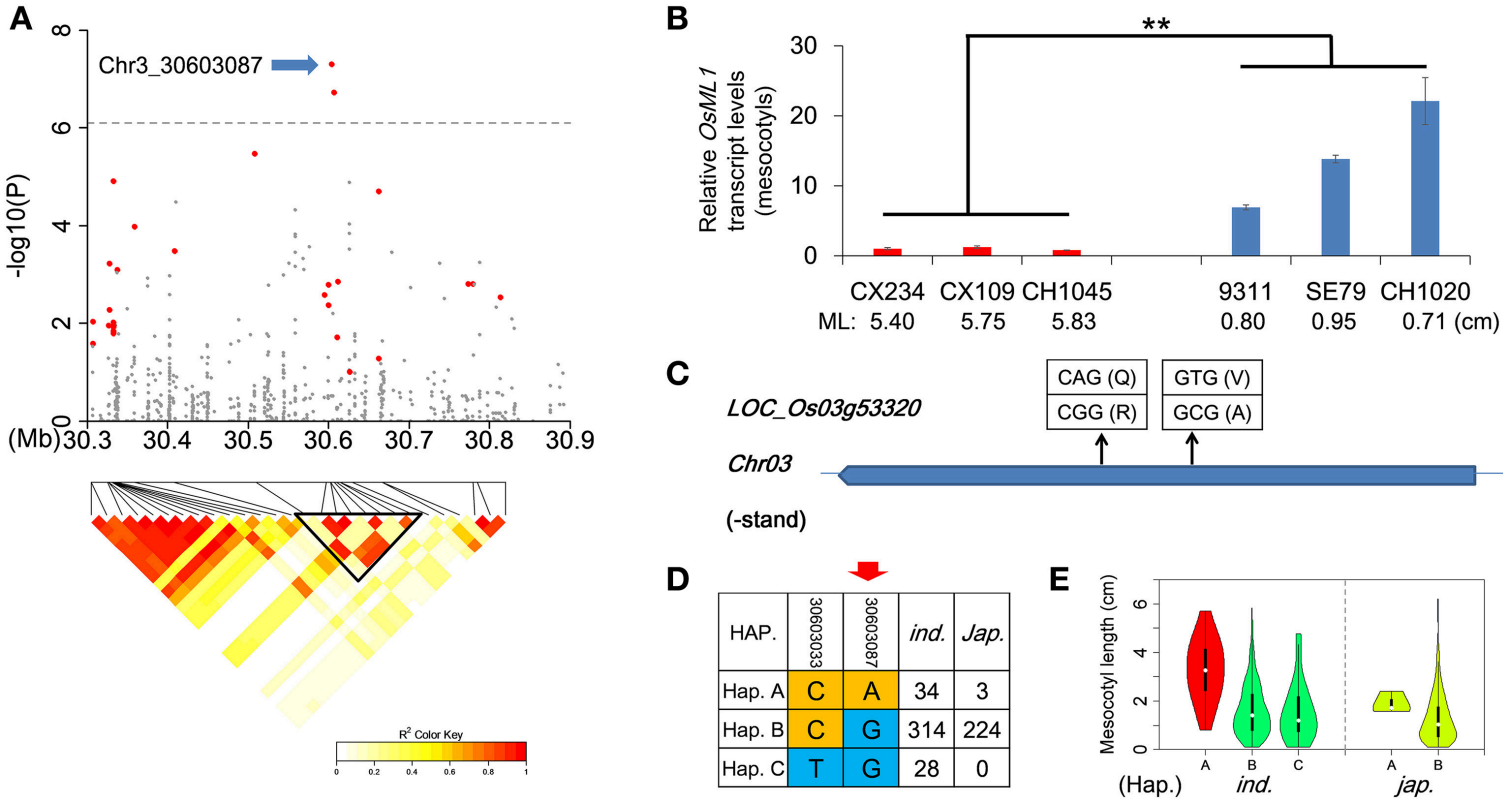
Identification of *OsML1* for mesocotyl length in *qFML3-2*. **(A)** Genome-wide association signals in the region at 30.3–30.9 Mb on chromosome 3 and LD heatmap (bottom) for 28 non-synonymous SNPs (colored red) with different alleles between parents of known bi-parental maps. Blue arrow indicates possible functional locus in *OsML1* (LOC_Os03g53320). Triangular block shows region with strong local LD (*r*^2^ > 0.6). **(B)** Expression analysis of *OsML1* in the mesocotyls from 6 accessions with long- and short-mesocotyls. Names and ML of accessions are plotted on the *X*-axis. Red and blue bars show long- and short-mesocotyl accessions, respectively. Data represent means ±s.d (*n* = 3). Asterisks represent significant differences between accessions with long- and short-mesocotyls (^**^p < 0.01, Student's *t* test). **(C)** Structure of *OsML1* showing non-synonymous SNPs and amino acid polymorphisms. **(D)** Haplotype analysis of two non-synonymous SNPs in *OsML1*. Red arrow indicates possible functional locus. **(E)** Comparison of ML among haplotypes of *OsML1* in *indica* and *japonica*. Accessions with haplotype colored red had significantly longer mesocotyls than members of haplotypes colored green.

By comparing Non-Syn GWAS to previous bi-parental mapping results (Redoña and Mackill, 1996b; Cai and Morishima, 2002; Cao et al., 2002; Ouyang et al., 2005; Huang C. et al., 2010; Lee H. et al., 2012; Lee H. S. et al., 2012; Eizenga et al., 2016), we confirmed 4 QTL for ML, including *qFML2-1, qFML3-1, qFML3-2*, and *qFML11-1*. Among them, QTL *qFML3-2* was previously identified for four times (Table S11). Based on high-density sequencing of four parental accessions in two of the bi-parental mapping studies (Table S11), we identified 28 non-synonymous SNPs with different alleles between the parents in the range of 300 kb around peak SNP (Chr3_30603087). LD analysis showed that 9 of 28 non-synonymous SNPs were in the same LD block with *r*^2^ > 0.6, including 2 non-synonymous SNPs (Chr3_30603087, Chr3_30606285) above the threshold with −log(*p*) values of 7.31 and 6.74, respectively (Figure 4A and (Table 1). The both loci were ESDSs with χ^2^-values of 20.72 and 19.12 in *indica* (Table 1). The analysis of association and linkage mapping and screening of ESDSs indicates that one or both non-synonymous SNPs could be functional loci located in candidate genes LOC_Os03g53320 and LOC_Os03g53340, respectively.

To confirm the functional gene for ML, we further investigated the relationship between ML and other polymorphic protein-coding differences in the two genes. There were three InDel (Chr3_30605444, Chr3_30605446 and Chr3_30606411) in the CDS of LOC_Os03g53340 in 621 accessions but none in LOC_Os03g53320 (Table S12). However, no significant difference in ML was detected between alleles of each InDel of LOC_Os03g53340. We also measured expression levels of the two candidate genes in mesocotyls using accessions with long- and short-mesocotyls by qRT-PCR. One (LOC_Os03g53320) showed more than 13.8-fold higher expression in short-mesocotyl accessions compared to long-mesocotyl accessions (Figure 4B), whereas the other (LOC_Os03g53340) showed no difference in expression levels in mesocotyls between long-mesocotyl and short-mesocotyl genotypes (Figure S10). Since there were not obvious differences in ML between alleles of 3 InDel as well as expression level between long- and short-mesocotyl accessions in LOC_Os03g53340, we suggest that LOC_Os03g53320 is the most likely functional gene for ML.

Here, we rename gene LOC_Os03g53320, which encodes a hypothetical protein, as *OsML1*. There were two non-synonymous SNPs in *OsML1* among the 621 accessions; only one of these (Chr3_30603087) showed an obvious signal in the integrated analysis (Figure 4C). Based on the two non-synonymous SNPs, we identified three haplotypes of *OsML1* in *indica* with distinct phenotypes. Accessions with haplotype A produced significantly longer mesocotyls than those containing haplotypes B or C (Figures 4D,E). Further sequence alignment between the long mesocotyl haplotype A and short mesocotyl haplotypes B and C showed that SNP (Chr3_30603087) could be the functional locus of *OsML1*.

### Exploration of candidate gene *OsML2*

**Figure 5:**
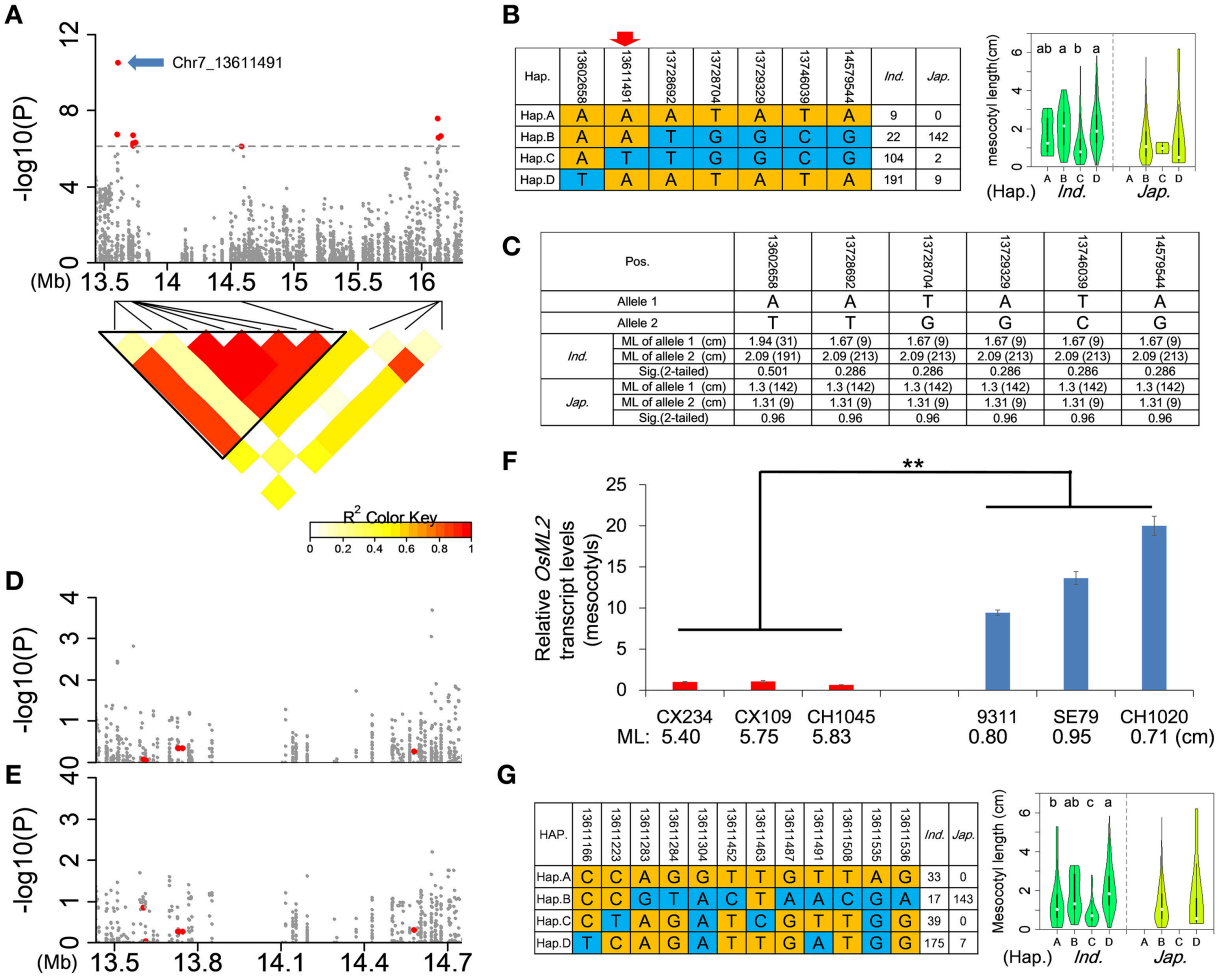
Identification of *OsML2* for mesocotyl length. **(A)** Genome-wide association signals (top) in the region 13.4–16.4 Mb on chromosome 7 and LD heatmap (bottom) of 10 non-synonymous SNPs above the threshold. A total of 10 red points show non-synonymous SNPs above threshold in *qFML7-2, qFML7-3* and *qFML7-4*, and blue arrow indicates possible functional locus in *OsML2* (LOC_Os07g24010). Triangular block (named Block 1) shows region with strong local LD (*r*^2^ > 0.6). **(B)** Haplotype analysis (left) of 7 non-synonymous SNPs above threshold in Block 1 and comparison of ML (right) among haplotypes in *indica* and *japonica*. Different letters above the violins indicate significant differences (*p* < 0.05) when analyzed by Duncan's test. Red arrow indicates possible functional locus. **(C)** Independent-sample *T*-tests for ML differences between alleles of each of the 6 non-synonymous SNPs in Block 1 using accessions carrying allele **(A)** of the peak signal (Chr7_13611491). Data in parentheses show number of accessions. Manhattan plots for ML in Block 1 using **(D)** 373 accessions and **(E)** 222 *indica* accessions carrying allele **(A)** of the peak signal. Red points show 6 non-synonymous SNPs in Block 1. **(F)** Expression analysis of *OsML2* in the mesocotyls from 6 accessions with long- and short-mesocotyls. Names and ML of accessions are plotted on the *X*-axis. Red and blue bars show long- and short-mesocotyl accessions, respectively. Data represent means ± s.d (*n* = 3). Asterisks represent significant differences between accessions with long- and short-mesocotyls (^**^p < 0.01, Student's *t* test). **(G)** Haplotype analysis (left) of 12 non-synonymous SNPs in *OsML2* and comparison of ML (right) among haplotypes of *OsML2* in *indica* and *japonica*, respectively. Different letters above the violins indicate significant differences (*p* < 0.05) detected by Duncan's test.

We focused on *qFML7-2* with the highest signal in the whole genome. In view of the short physical distance covering *qFML7-2, qFML7-3*, and *qFML7-4* we redefined the QTL region based on local LD. As indicated in the LD heatmap seven non-synonymous SNPs above threshold in *qFML7-2* and *qFML7-3* are in the same LD block (named as Block 1) (Figure 5A). We also found strong LDs with *r*^2^ of ≈0.9 among the seven SNPs, except for the peak non-synonymous SNP (Chr7_13611491) with *r*^2^ of ≈0.5. These results indicated that one of the seven non-synonymous SNPs within Block 1 could be the functional locus for ML.

After comparisons with previous bi-parental mapping studies we established that the QTL was a new locus for ML. We checked the alleles of all 7 non-synonymous SNPs between the parental accessions of the previous bi-parental mapping populations (Table S13) and no allelic difference could explain why this QTL was not identified in previous mapping studies.

To search for functional SNPs and genes for ML in Block 1, we performed a sequence alignment analysis of the 7 non-synonymous SNPs in the LD block. In the full population there were four main haplotypes (A–D) involving more than three accessions. Three (B, C, and D) and all four haplotypes were detected in *japonica* and *indica*, respectively (Figure 5B). Accessions carrying haplotype C had shorter ML than those carrying B or D, with significant differences in *indica*. The peak non-synonymous SNP (Chr7_13611491) was the only locus with a consistent allele (A) in long mesocotyl haplotypes B and D, whereas allele (T) was present in short mesocotyl haplotype C (Figure 5B). We suggest that the peak non-synonymous SNP might be the functional locus for ML in Block 1.

To check the inference we divided the population into groups carrying alleles (A) and (T) of the peak locus, and re-examined the relationship between ML and each of other 6 non-synonymous SNPs above threshold in Block 1. In 222 and 151 accessions carrying allele (A) in *indica* and *japonica*, respectively, there was no significant difference in ML between alleles of each non-synonymous SNP (Figure 5C). In 104 and 2 accessions carrying allele (T) in *indica* and *japonica*, we detected no allelic diversity in the other 6 non-synonymous SNPs (Figure 5B). We performed another complementary GWAS using accessions carrying allele (A) of the peak SNP in the full population and *indica*, respectively. There was no signal for ML in Block 1, and the 6 non-synonymous SNPs showed *p*-values < −log(*p*) = 1 in the full population and *indica* (Figures 5D,E). These results showed that the other 6 non-synonymous SNPs were not functional loci associated with ML, despite the higher −log(*p*) values in the original Non-Syn GWAS of the full population and *indica* (Tables 1 and Table S8). We suggest that the signals in the other 6 non-synonymous SNPs were produced in accessions carrying allele (T) of the peak SNP (Chr7_13611491).

We checked the expression level of the candidate LOC_Os07g24010 including non-synonymous SNP Chr7_13611491. There was about a 15.9-fold higher expression in short-mesocotyl accessions compared to long-mesocotyl accessions (Figure 5F). We renamed LOC_Os07g24010 as *OsML2*; it also encoded a hypothetical protein. There were 12 non-synonymous SNPs in *OsML2*. Among them, six (13611166, 13611223, 13611304, 13611463, 13611491, and 13611535) showed high signals with −log(*p*) > 4 in the Non-Syn GWAS using the full population (Table S14). Based on the 12 non-synonymous SNPs in *OsML2*, four and two haplotypes were identified in *indica* and *japonica*, respectively. There were clear differences in ML among accessions carrying haplotypes A, B, C, or D (Figure 5G). The results showed that *OsML2* was an important expressed gene in regulating mesocotyl elongation, and several non-synonymous SNPs were associated with ML.

### Cross-validation of two genes for mesocotyl length

**Figure 6:**
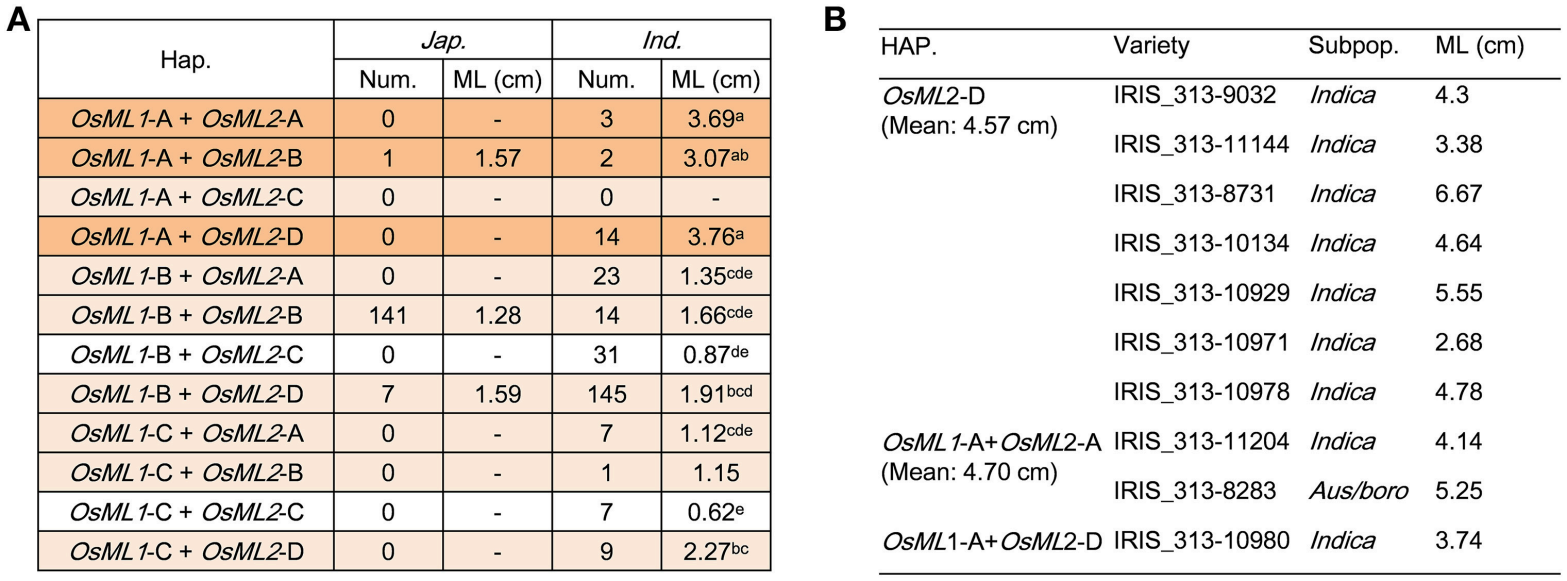
Functional validation of haplotype combinations of *OsML1* and *OsML2*. **(A)** ML of different combinations of haplotypes in *OsML1* and *OsML2*. Different letters indicate significant differences (*p* < 0.05) detected by one-way ANOVA. **(B)** Cross-validation by phenotyping of 10 accessions representing different genotypes of *OsML1* and *OsML2*.

For efficient utilization of *OsML1* and *OsML2* in breeding for deep-sowing tolerance, it is important to uncover the genotypic effect of each haplotype and the combined haplotypes of two genes. Based on the above sequence alignment analysis of each gene, we focused on *OsML1*-A, *OsML2*-A, *OsML2*-B, and *OsML2*-D as long ML haplotypes, whereas *OsML1*-B, *OsML1*-C, and *OsML2*-C were considered to be short ML haplotypes. Among 12 possible allelic combinations of the two genes, eleven could be detected in 621 rice accessions. There were more genetic diversity in *indica*, including 11 allelic combinations, but only 3 allelic combinations were identified in *japonica* (Figure 6A). Considering the complexity of population structure and genetic background, we performed statistical analysis in *indica* by one-way ANOVA. The MLs were the shortest in accessions without long ML genotypes, *OsML1*-C + *OsML2*-C (0.62 cm) and *OsML1*-B + *OsML2*-C (0.87 cm), followed by accessions with only one long ML genotypes, *OsML1*-C + *OsML2*-A (1.12 cm), *OsML1*-C + *OsML2*-B (1.15 cm), *OsML1*-B + *OsML2*-A (1.35 cm), *OsML1*-B + *OsML2*-B (1.66 cm), *OsML1*-B + *OsML2*-D (1.91 cm), *OsML1*-C + *OsML2*-D (2.27 cm), while accessions with both long ML genotypes had longest ML, *OsML1*-A + *OsML2*-B (3.07 cm), *OsML1*-A + *OsML2*-A (3.69 cm), and *OsML1*-A + *OsML2*-D (3.76 cm) (Figure 6A). Haplotypes *OsML1*-A and *OsML2*-D formed the best combination with longest ML and should be the best genotype for use in molecular marker assisted breeding for long ML.

For validation, we identified accessions with long ML haplotypes for each gene from 3,024 cultivated rice accessions (Li J. Y. et al., 2014; Alexandrov et al., 2015). Three hundred and sixty seven and 982 accessions were detected with *OsML1*-A and *OsML2*-D, respectively, and 110 had both alleles. We selected 7 lines with *OsML2*-D, 2 lines with *OsML1*-A and *OsML2*-A and 1 line with *OsML1*-A and *OsML2*-D, and grew them in a plastic box with 10 cm of soil cover. The mean MLs were 4.57, 4.70, and 3.74 cm, respectively (Figure 6B). Unfortunately no seed of other accessions was available, but the results supported the hypothesis that *OsML1* and *OsML2* are functional genes and that the long ML haplotypes acting in an additive manner can be used in molecular marker assisted breeding. Additionally, we demonstrated a powerful strategy for efficient cloning of complex trait QTL that combines Non-Syn GWAS, known linkage map information, allelic frequencies differences between phenotypic pools, expression analyses and haplotype analyses.

### Evidence of positive selection on *OsML1* in *Japonica*

To explore whether the two genes were targeted by natural or human selection during rice domestication, we performed signature identification of selection using 621 cultivated and 446 wild rice accessions (Huang et al., 2012a). We extracted the sequencing data including the gene and a 2 kb promoter region, respectively, within *OsML1* and *OsML2*. Selective signal scan were performed within the both genes using the ratio of the genetic diversity in wild rice to that in *indica* and *japonica* (π_W_/π_I_ and π_W_/π_J_), respectively (Table 2). High selective signal was detected within *OsML1* in *japonica* (π_W_/π_J_ = 4.59). The Tajima's *D* was lowest at the value of −2.19, suggesting strong positive selection across *OsML1* in *japonica*.

**Table 2:** Nucleotide diversity and Tajima's *D* Test.

**Gene_Population**	**π**	**Tajima's *D***
*OsML1*_Wild	0.00224	−0.65
*OsML1*_Cultivated	0.00301	0.20
*OsML1*_*Ind*.	0.00365	1.97
*OsML1*_*Jap*.	0.00049	−2.20
*OsML2*_Wild	0.0061	1.31
*OsML2*_Cultivated	0.01671	1.27
*OsML2*_*Ind*.	0.01093	0.30
*OsML2*_*Jap*.	0.00604	−1.02

## Discussion

### Mesocotyl elongation is a key trait for deep-sowing tolerance

Rice accessions for dry direct-seeding need have the ability to withstand deep planting for access to moisture or to tolerate variable planting depth associated with machine sowing. Many studies have been carried out to identify deep-sowing tolerant cereal genotypes and to examine the relationship between mesocotyl and/or coleoptile length and deep-sowing tolerance (Turner et al., 1982; Kirby, 1993; Luo et al., 2007; Chung, 2010; Wu et al., 2015; Lu et al., 2016). Here a diverse set of 621 cultivars was tested for deep-sowing tolerance under field conditions and in subsequent depth-of-sowing experiments in plastic boxes. Our results, like previous studies, confirmed two points: (1) ML is induced by deeper soil covering (Wu et al., 2015; Lu et al., 2016), (2) and variation in ML fits a negative binomial distribution and there are few elite cultivated rice genotypes with long mesocotyls (Luo et al., 2007). Moreover, our results showed that ER was not affected greatly until the sowing depth reached 3 cm. Therefore, we suggest that new accessions for dry direct-seeding should have ML of more than 3 cm.

Comparison of ML between the two main rice subgroups showed that average ML in *indica* was significantly longer than in *japonica*. Given that *indica* rice was originally developed from crosses between *japonica* and local wild rice (Huang et al., 2012a) and that weedy and wild rice have longer ML (Cai and Morishima, 2002; Chung, 2010; Eizenga et al., 2016), we suggest that short mesocotyls are probably the result of an evolutionary change due to the long period of traditional rice cultivation by transplantation. Weedy and wild rice could be elite genetic resources for solving the problem of poor seedling establishment in direct-seeded rice (Chung, 2010). However, in the present work we also identified several germplasm with long mesocotyls and good seedling emergence under deep sown conditions and those elite germplasm can be used as parents in breeding rice accessions for dry direct-seeding.

### Non-syn GWAS was conducive in identifying functional loci and alleles

GWAS has become a common method in searching for candidate genes underlying target traits in rice (Huang X. et al., 2010; Huang et al., 2012b; Si et al., 2016; Yano et al., 2016). The Mixed Linear Model (MLM) with PC and kinship is efficient in decreasing false positives due to population structure and relationships among individuals (Yu et al., 2006). However, for complex traits affected by population structure, MLM weakens the real association and reduces signal strength of known genes. CMLM improves the statistical power by using group kinship (Zhang et al., 2010a). Constructing a trait-specific kinship derived from weighted SNP analysis is another strategy to improve MLM, as achieved by SUPER and FarmCPU (Wang et al., 2014; Liu et al., 2016). We derived two sets of SNPs (groups I and II) from 15 million polymorphisms according to annotated gene locations and possible influence on gene function, and then we obtained 572,511 non-synonymous SNPs and 1,801,421 SNPs located in the 5′ sequences of genes (≤1 Kb upstream of the first ATG) by this approach. Finally, we performed and compared GWAS using these groups to search for candidate genes affecting ML. this provides an alternative solution for constructing a trait-specific kinship derived from polymorphisms that can be screened out according to biological function of target traits. Additionally, this greatly reduced the computational burden by performing GWAS using these SNPs that accounted for amino acid sequence and expression.

There were two differences between Non-Syn GWAS and GWAS using a random sample of genetic markers (group III), one was the loci, the other was PC and kinship matrix derived from these loci. In our studies, we assessed their impact on the GWAS results. By comparison of GWAS between ML and SNPs in group I using different PC and kinship, Non-Syn GWAS detected the same SNPs for ML as GWAS with PC and kinship derived from group III. The results suggested that CMLM using PC and kinship derived from group I had same efficiency as CMLM using PC and kinship from group III. Further comparison of Non-Syn GWAS and GWAS using group III indicated that there were less loci in QTL regions (local LD block of peak SNP) identified by Non-Syn GWAS. Thus, we were able to quickly and conveniently identify functional loci by the integrated analysis without confounding by other loci with little likelihood of biological function.

### *OsML1* and *OsML2* are natural variants for mesocotyl length, and could be useful in breeding for deep-sowing tolerance

Four of 13 QTLs in Non-Syn GWAS of the full population overlapped with loci identified in previous linkage mapping studies (Cai and Morishima, 2002; Ouyang et al., 2005; Huang C. et al., 2010; Lee H. S. et al., 2012). Among them, *qFML3-2* located in chromosome 3 was previously detected four times in bi-parental mapping studies. However, none QTL in Non-Syn GWAS overlapped with association signal in previous GWAS (Wu et al., 2015; Lu et al., 2016). The most likely reason for this finding was that different depths of soil cover were applied in these GWAS (Wu et al., 2015; Lu et al., 2016).

Interestingly, both genes showed higher expression in short-mesocotyl accessions compared to long-mesocotyl accessions. Furthermore, long ML haplotypes of both genes (*OsML1*-A, *OsML2*-A, *OsML2*-B, and *OsML2*-D) could be loss-of-function or partial loss-of-function variations. Nucleotide diversity analysis and Tajima's *D* Test showed that *OsML1* was strongly directly selected in *japonica* accessions. These results supported that short ML was a target of selection during the long term process of conversion from wild rice to *japonica* under the traditional transplanting system.

Sequence alignment of two genes also showed that there was less genetic diversity of *OsML1* and *OsML2* in *japonica*, and most of *japonica* rice had haplotypes *OsML1*-B and *OsML2*-B associated with short mesocotyls. However, there were significant differences among *OsML1* and *OsML2* haplotypes in *indica*. Superior haplotypes with long mesocotyls (*OsML1*-A and *OsML2*-D) can be used for molecular marker assistant selection, and those two genes can also be further cloned and analyzed, then used for gene transformation and breeding of rice accessions for deep-sowing tolerance.

## Author contributions

YZ, WZ, CJ, JL, and ZL designed the research, performed most of experiments and analyzed the data. XiaW, HX, ET, ZY, YC, and XinW performed part of the experiments. JX, YP, MR, and HZ conceived and supervised the project. YZ and ZL conceived the experiment and wrote the manuscript.

### Conflict of interest statement

The authors declare that the research was conducted in the absence of any commercial or financial relationships that could be construed as a potential conflict of interest.
