# Supplementary material for: Genetic Architecture and Candidate Genes for Deep-Sowing Tolerance in Rice Revealed by Non-syn GWAS
Source: Front Plant Sci. 2018 Mar 16;9:332. doi: 10.3389/fpls.2018.00332 (PMC5864933; doi:10.3389/fpls.2018.00332)
Supplement: Supplementary file 1 [file Table1.DOCX]

**Table S1. Analysis of mesocotyl length variation between duplicates.**

Paired samples statistics

| Duplicate | Mean | N | Std. deviation | Std. error of mean |
| --- | --- | --- | --- | --- |
| Duplicate 1 | 1.60 | 621 | 1.23 | .049 |
| Duplicate 2 | 1.61 | 621 | 1.31 | .053 |

Paired Sample Correlations

|  | N | Correlation | Sig. |
| --- | --- | --- | --- |
| Duplicate 1 & duplicate 2 | 621 | .599 | .000 |

Paired sample tests

|  | Paired differences | | | | | t | df | Sig. (2-tailed) |
| --- | --- | --- | --- | --- | --- | --- | --- | --- |
|  | Mean | Std. D | Std. E. M | 95% C. I. D | |  |  |  |
|  |  |  |  | Lower | Upper |  |  |  |
| Duplicate 1 - 2 | -.017 | 1.14 | .046 | -.10 | 0.07 | -.381 | 620 | 0.704 |
