# Supplementary material for: Genetic Architecture and Candidate Genes for Deep-Sowing Tolerance in Rice Revealed by Non-syn GWAS
Source: Front Plant Sci. 2018 Mar 16;9:332. doi: 10.3389/fpls.2018.00332 (PMC5864933; doi:10.3389/fpls.2018.00332)
Supplement: Supplementary file 2 [file Table2.DOCX]

**Table S2. Comparison of the mesocotyl length variation between *japonica* and *indica*.**

| Subspecies | N | Mean | Std. D | Std. E. M. | t | df | Sig. (2-tailed) | Mean D. | Std. E. D. |
| --- | --- | --- | --- | --- | --- | --- | --- | --- | --- |
| *Japonica* | 231 | 1.32 | 1.01 | 0.07 | -5.12 | 541 | .000 | -0.46 | 0.09 |
| *Indica* | 390 | 1.78 | 1.18 | 0.06 |  |  |  |  |  |
