# Supplementary material for: Genetic Architecture and Candidate Genes for Deep-Sowing Tolerance in Rice Revealed by Non-syn GWAS
Source: Front Plant Sci. 2018 Mar 16;9:332. doi: 10.3389/fpls.2018.00332 (PMC5864933; doi:10.3389/fpls.2018.00332)
Supplement: Supplementary file 3 [file Table3.DOCX]

**Table S3. Summary of accessions with mesocotyl length of more than 4 cm.**

| Subpopulation | accession | PC1 | PC2 | ML (cm) | Plant height (cm) | Name | Country of origin |
| --- | --- | --- | --- | --- | --- | --- | --- |
| *Ind.* | CH1045 | 389 | -37 | 5.83 | 107.8 | Jaibattey | India |
|  | CX472 | 366 | -32 | 5.71 | 90.6 | 838 | - |
|  | CX234 | 394 | -8 | 5.4 | 87.8 | IR62266-42-6-2 | - |
|  | CX153 | 382 | -23 | 5.29 | 127.2 | Jalmagna | India |
|  | CX368 | 132 | -216 | 5.22 | 104.1 | N22 | - |
|  | CX240 | 419 | -42 | 4.96 | 105.9 | Carijo | - |
|  | CX8 | 340 | -48 | 4.84 | 87.4 | Chenghui 448 | China |
|  | CH1077 | 119 | -185 | 4.82 | 152.4 | Weedy rice 13 | Nepal |
|  | CX338 | 428 | 9 | 4.77 | 83 | B5-10 | - |
|  | CX120 | 318 | -109 | 4.7 | 109 | TKM 6 | India |
|  | CH1044 | 406 | 27 | 4.68 | 83.1 | Dumai | India |
|  | CX337 | 434 | 13 | 4.57 | 85.5 | B5 | China |
|  | CX130 | 333 | -13 | 4.44 | 118.1 | CHIPDA | India |
|  | CX197 | 434 | 28 | 4.41 | 94.2 | 084 | - |
|  | CX301 | 364 | -24 | 4.27 | 98.3 | Qianhui 875 | China |
|  | CX185 | 365 | -20 | 4.15 | 82.2 | Zhonghui 8006 | - |
|  | CX98 | 390 | -48 | 4.13 | 116.4 | Doddabyranella | - |
|  | CX313 | 362 | -28 | 4.11 | 95.2 | R106 | China |
|  | CX382 | 405 | -24 | 4.11 | 86.6 | Kogomg 1-1 | - |
|  | CH1046 | 424 | -7 | 4.08 | 127.7 | Ngatsin | India |
|  | CX131 | 437 | 31 | 4.08 | 98.3 | ZALE | Myanmar |
|  | CH1087 | 319 | -95 | 4.04 | 121.5 | J34 | Madgascar |
|  | CH1162 | 401 | -9 | 4.04 | 72 | Xiaohonggu | China |
| *Jap.* | CX242 | -114 | -266 | 6.19 | 139.7 | IR47686-4-4-B-1 | - |
|  | CX109 | -586 | -185 | 5.75 | 120.9 | Dacca 6 | Philippines |
|  | CH1119 | -685 | -133 | 4.13 | 106.2 | Dandongludao | China |
|  | CX106 | -599 | -485 | 4.02 | 118.8 | SAL BUI BAO | Vietnam |
