# Supplementary material for: Genetic Architecture and Candidate Genes for Deep-Sowing Tolerance in Rice Revealed by Non-syn GWAS
Source: Front Plant Sci. 2018 Mar 16;9:332. doi: 10.3389/fpls.2018.00332 (PMC5864933; doi:10.3389/fpls.2018.00332)
Supplement: Supplementary file 4 [file Table4.DOCX]

**Table S4. Paired sample correlations between ML and plant height.**

|  | N | Correlation | Sig. |
| --- | --- | --- | --- |
| ML & plant height in full pop. | 617 | .230 | .000 |
| ML & plant height in *indica* | 387 | .189 | .000 |
| ML & plant height in *japonica* | 230 | .385 | .000 |
