# Supplementary material for: Genetic Architecture and Candidate Genes for Deep-Sowing Tolerance in Rice Revealed by Non-syn GWAS
Source: Front Plant Sci. 2018 Mar 16;9:332. doi: 10.3389/fpls.2018.00332 (PMC5864933; doi:10.3389/fpls.2018.00332)
Supplement: Supplementary file 5 [file Table5.DOCX]

**Table S5. Accessions in polar pools in *indica* and *japonica*.**

| Subspecies | Polar pool | ML (cm) | PC1 | PC2 | Accession |
| --- | --- | --- | --- | --- | --- |
| *Ind.* | Low pool | 0.10 | 313 | -31 | CH1074 |
|  |  | 0.10 | 415 | 81 | CX10 |
|  |  | 0.13 | 397 | 16 | CH1107 |
|  |  | 0.14 | 425 | 14 | CX2 |
|  |  | 0.16 | 448 | 26 | CX349 |
|  |  | 0.17 | 411 | -5 | CX122 |
|  |  | 0.18 | 447 | 42 | CX162 |
|  |  | 0.20 | 436 | -1 | CX25 |
|  |  | 0.20 | 445 | 21 | CH1210 |
|  |  | 0.20 | 419 | 6 | CX360 |
|  |  | 0.22 | 442 | 31 | CX79 |
|  |  | 0.24 | 363 | -2 | CH1225 |
|  |  | 0.25 | 394 | 28 | CX361 |
|  |  | 0.25 | 450 | 44 | CX508 |
|  |  | 0.25 | 464 | 67 | CH1208 |
|  |  | 0.26 | 338 | -35 | CH1056 |
|  |  | 0.29 | 423 | 36 | CH1205 |
|  |  | 0.29 | 431 | 14 | CX202 |
|  |  | 0.30 | 448 | 56 | CH1204 |
|  |  | 0.31 | 437 | 54 | CH1277 |
|  | High pool | 4.04 | 401 | -9 | CH1162 |
|  |  | 4.08 | 424 | -7 | CH1046 |
|  |  | 4.08 | 437 | 31 | CX131 |
|  |  | 4.11 | 362 | -28 | CX313 |
|  |  | 4.11 | 405 | -24 | CX382 |
|  |  | 4.13 | 390 | -48 | CX98 |
|  |  | 4.15 | 365 | -20 | CX185 |
|  |  | 4.27 | 364 | -24 | CX301 |
|  |  | 4.41 | 434 | 28 | CX197 |
|  |  | 4.44 | 333 | -13 | CX130 |
|  |  | 4.57 | 434 | 13 | CX337 |
|  |  | 4.68 | 406 | 27 | CH1044 |
|  |  | 4.70 | 318 | -109 | CX120 |
|  |  | 4.77 | 428 | 9 | CX338 |
|  |  | 4.84 | 340 | -48 | CX8 |
|  |  | 4.96 | 419 | -42 | CX240 |
|  |  | 5.29 | 382 | -23 | CX153 |
|  |  | 5.40 | 394 | -8 | CX234 |
|  |  | 5.71 | 366 | -32 | CX472 |
|  |  | 5.83 | 389 | -37 | CH1045 |
| *Jap.* | Low pool | 0.10 | -873 | 187 | CH1305 |
|  |  | 0.14 | -585 | -161 | CX1 |
|  |  | 0.16 | -689 | -89 | CH1006 |
|  |  | 0.20 | -863 | 174 | CH1071 |
|  |  | 0.20 | -590 | -466 | CH1027 |
|  |  | 0.22 | -851 | 156 | CX116 |
|  |  | 0.23 | -867 | 175 | CH1008 |
|  |  | 0.24 | -765 | 164 | CH1098 |
|  |  | 0.25 | -823 | 192 | CX251 |
|  |  | 0.26 | -760 | 174 | CX299 |
|  |  | 0.27 | -742 | 114 | CH1097 |
|  |  | 0.29 | -843 | 146 | CX529 |
|  |  | 0.29 | -785 | 172 | CX545 |
|  |  | 0.30 | -859 | 164 | CX181 |
|  |  | 0.30 | -836 | 166 | CX396 |
|  |  | 0.31 | -826 | 113 | CX527 |
|  |  | 0.31 | -815 | 153 | CH1026 |
|  |  | 0.31 | -790 | 164 | CX546 |
|  |  | 0.31 | -776 | 110 | CH1291 |
|  |  | 0.31 | -769 | 101 | CH1122 |
|  | High pool | 2.63 | -635 | -404 | CH1067 |
|  |  | 2.64 | -609 | 55 | CX138 |
|  |  | 2.89 | -602 | -409 | CH1091 |
|  |  | 2.95 | -761 | -39 | CH1023 |
|  |  | 2.96 | -683 | 0 | CX384 |
|  |  | 2.99 | -745 | 138 | CX380 |
|  |  | 2.99 | -734 | -40 | CH1184 |
|  |  | 3.01 | -543 | -488 | CX248 |
|  |  | 3.02 | -486 | -19 | CX282 |
|  |  | 3.20 | -497 | 25 | CX441 |
|  |  | 3.21 | -645 | -238 | CH1240 |
|  |  | 3.21 | -570 | -479 | CX113 |
|  |  | 3.21 | -464 | -391 | CX243 |
|  |  | 3.48 | -648 | -240 | CH1296 |
|  |  | 3.77 | -834 | 99 | CH1054 |
|  |  | 3.93 | -710 | -146 | CH1257 |
|  |  | 3.95 | -673 | -252 | CH1059 |
|  |  | 4.02 | -599 | -485 | CX106 |
|  |  | 4.13 | -685 | -133 | CH1119 |
|  |  | 5.75 | -586 | -185 | CX109 |
