# Supplementary material for: Genetic Architecture and Candidate Genes for Deep-Sowing Tolerance in Rice Revealed by Non-syn GWAS
Source: Front Plant Sci. 2018 Mar 16;9:332. doi: 10.3389/fpls.2018.00332 (PMC5864933; doi:10.3389/fpls.2018.00332)
Supplement: Supplementary file 6 [file Table6.DOCX]

**Table S6. Summary of SNPs associated with mesocotyl length by GWAS using CMLM and group II in full population.**

| QTL | Gene | Position | -log(*p*)^a^ | -log(*p*)^b^ | -log(*p*)^c^ | SNP variation | Amino acid variation | MAF | Functional annotation |
| --- | --- | --- | --- | --- | --- | --- | --- | --- | --- |
| *qFML1-1* | LOC_Os01g66510 | Chr1_38626758 | 7.13 | 8.74 | 7.1 | - | - | 0.01 | MLO domain containing protein, putative, expressed |
| *qFML2-1* | LOC_Os02g07420 | Chr2_3832764 | 6.71 | 7.72 | 6.71 | - | - | 0.02 | Splicing factor, arginine/serine-rich 12, putative, expressed |
|  | LOC_Os02g07495 | Chr2_3873623 | 6.85 | 8.11 | 6.93 | - | - | 0.02 | Expressed protein |
|  |  | Chr2_3873651 | 6.85 | 8.69 | 7.01 | - | - | 0.01 |  |
|  |  | Chr2_3873861 | 7.28 | 9.08 | 7.35 | - | - | 0.01 |  |
|  | LOC_Os02g07650 | Chr2_3970604 | 8.26 | 10.3 | 8.42 | - | - | 0.01 | Zinc-binding protein, putative, expressed |
| *qFML3-1* | LOC_Os03g52910 | Chr3_30348618 | 6.93 | 12.32 | 7.04 | - | - | 0.07 | Uncharacterized UPF0114 domain containing protein, expressed |
|  | LOC_Os03g53180 | Chr3_30508698 | 6.82 | 11.77 | 6.84 | - | - | 0.07 | Expressed protein |
|  | LOC_Os03g53280 | Chr3_30582925 | 7.9 | 14.49 | 8.05 | - | - | 0.08 | WD domain containing protein, putative, expressed |
|  | LOC_Os03g53310 | Chr3_30600087 | 6.79 | 11.7 | 6.81 |  |  | 0.07 | Emp24/gp25L/p24 family protein, putative, expressed |
|  | LOC_Os03g53320 | Chr3_30603087 | 7.23 | 12.39 | 7.24 | G/A | A/V | 0.07 | Hypothetical protein |
| *qFML7-1* | LOC_Os07g17370 | Chr7_10256994 | 7.66 | 6.65 | 7.47 | - | - | 0.04 | Expressed protein |
| *qFML7-2* | LOC_Os07g23740 | Chr7_13398939 | 7.37 | 9.62 | 7.12 | - | - | 0.38 | Sterol 3-beta-glucosyltransferase, putative, expressed |
|  | LOC_Os07g23960 | Chr7_13555251 | 6.93 | 8.87 | 6.58 | - | - | 0.17 | Transferase family protein, putative, expressed |
|  | LOC_Os07g23990 | Chr7_13602658 | 7.22 | 9.92 | 7.04 | A/T | M/L | 0.37 | Tetratricopeptide repeat domain containing protein, putative, expressed |
|  | LOC_Os07g24000 | Chr7_13607033 | 7.6 | 10.39 | 7.4 | - | - | 0.36 | AWPM-19-like membrane family protein, putative, expressed |
|  | LOC_Os07g24010 | Chr7_13611491 | 11.2 | 15.5 | 10.35 | A/T | S/T | 0.26 | Hypothetical protein |
|  | LOC_Os07g24030 | Chr7_13624700 | 7.86 | 11.26 | 7.55 | - | - | 0.42 | Hypothetical protein |
|  | LOC_Os07g24050 | Chr7_13637538 | 8.44 | 11.84 | 8.19 | - | - | 0.41 | Carboxyl-terminal proteinase, putative, expressed |
|  |  | Chr7_13637833 | 7.3 | 10.35 | 7.06 | - | - | 0.41 |  |
|  | LOC_Os07g24140 | Chr7_13699476 | 8.02 | 10 | 7.81 | - | - | 0.45 | Hypothetical protein |
|  |  | Chr7_13699658 | 8.68 | 10.72 | 8.43 | - | - | 0.45 |  |
|  |  | Chr7_13699676 | 8.28 | 10.5 | 8.06 | - | - | 0.45 |  |
|  |  | Chr7_13699956 | 7.26 | 8.88 | 7.03 | - | - | 0.45 |  |
|  | LOC_Os07g24150 | Chr7_13706450 | 7.33 | 8.98 | 7.06 | - | - | 0.46 | Expressed protein |
|  |  | Chr7_13706973 | 8.88 | 10.56 | 8.67 | - | - | 0.45 |  |
|  | LOC_Os07g24170 | Chr7_13728692 | 7.13 | 9.99 | 6.95 | T/A | N/K | 0.39 | Expressed protein |
|  | LOC_Os07g24190 | Chr7_13746039 | 6.73 | 9.34 | 6.53 | C/T | M/I | 0.38 | CESA3 - cellulose synthase, expressed |
|  | LOC_Os07g24200 | Chr7_13769017 | 6.82 | 9.56 | 6.67 | - | - | 0.39 | Expressed protein |
|  | LOC_Os07g24230 | Chr7_13780752 | 6.83 | 6.43 | 6.63 | - | - | 0.49 | Integral membrane transporter family protein, putative, expressed |
|  |  | Chr7_13781153 | 7.59 | 7.14 | 7.41 | - | - | 0.5 | #N/A |
|  | LOC_Os07g24330 | Chr7_13843334 | 6.8 | 6.4 | 6.63 | - | - | 0.49 | Conserved hypothetical protein |
| *qFML7-3* | LOC_Os07g25060 | Chr7_14303315 | 7.48 | 6.91 | 7.31 | - | - | 0.49 | Thionin-like peptide, putative, expressed |
| *qFML7-4* | LOC_Os07g25480 | Chr7_14593993 | 6.91 | 9.47 | 6.7 | - | - | 0.38 | Expressed protein |
|  | LOC_Os07g25490 | Chr7_14601372 | 6.99 | 9.55 | 6.76 | - | - | 0.38 | Expressed protein |
| *qFML7-5* | LOC_Os07g27610 | Chr7_16128799 | 6.79 | 12.16 | 6.4 | - | - | 0.18 | Expressed protein |
|  |  | Chr7_16128978 | 7.8 | 12.97 | 7.54 | - | - | 0.43 |  |
|  |  | Chr7_16129009 | 6.72 | 11.86 | 6.34 | - | - | 0.18 |  |
|  |  | Chr7_16129307 | 7.63 | 13.02 | 7.41 | - | - | 0.44 |  |
|  |  | Chr7_16129346 | 7.37 | 12.37 | 7.07 | - | - | 0.44 |  |
|  |  | Chr7_16129890 | 7.84 | 13.23 | 7.56 | G/A | R/Q | 0.44 |  |
|  | LOC_Os07g27630 | Chr7_16135146 | 6.88 | 12.21 | 6.5 | G/A | S/L | 0.19 | Expressed protein |
|  |  | Chr7_16136034 | 7.58 | 12.69 | 7.3 | - | - | 0.44 |  |
|  |  | Chr7_16136035 | 7.55 | 12.61 | 7.26 | - | - | 0.44 |  |
|  |  | Chr7_16136131 | 7.66 | 13.01 | 7.43 | - | - | 0.44 |  |
|  | LOC_Os07g27650 | Chr7_16144452 | 7.85 | 12.7 | 7.51 | - | - | 0.43 | Expressed protein |
|  |  | Chr7_16144627 | 7.33 | 12.03 | 7.04 | - | - | 0.43 |  |
|  | LOC_Os07g27680 | Chr7_16151805 | 7 | 12.39 | 6.65 | T/C | T/A | 0.46 | Expressed protein |
| *qFML7-6* | LOC_Os07g31480 | Chr7_18672489 | 6.94 | 9.99 | 6.82 | - | - | 0.11 | Expressed protein |
| *qFML7-7* | LOC_Os07g39660 | Chr7_23772063 | 7.19 | 11.27 | 7.13 | - | - | 0.24 | Hypothetical protein |
|  |  | Chr7_23772066 | 7.14 | 11.16 | 7.09 | - | - | 0.24 |  |
| *qFML11-1* | LOC_Os11g10920 | Chr11_6031396 | 8.01 | 9.42 | 8.18 | A/C | V/G | 0.01 | Carboxyl-terminal proteinase, putative, expressed |
|  | LOC_Os11g10990 | Chr11_6065939 | 6.82 | 7.91 | 6.92 | C/T | G/R | 0.01 | Heat shock protein DnaJ, putative, expressed |

^a^, -log(*p*) are association signals of CMLM using PC and kinship derived from group II.

^b^, -log(*p*) are association signals of GLM using PC derived from group II.

^c^, -log(*p*) are association signals of CMLM using PC and kinship derived from group III.
