# Supplementary material for: Genetic Architecture and Candidate Genes for Deep-Sowing Tolerance in Rice Revealed by Non-syn GWAS
Source: Front Plant Sci. 2018 Mar 16;9:332. doi: 10.3389/fpls.2018.00332 (PMC5864933; doi:10.3389/fpls.2018.00332)
Supplement: Supplementary file 7 [file Table7.DOCX]

**Table S7. Summary of SNPs associated with mesocotyl length by GWAS using CMLM and group Ⅲ in full population.**

| QTL | Gene | Position | -log(*p*)^a^ | -log(*p*)^b^ | SNP variation | Amino acid variation | MAF | Functional annotation |
| --- | --- | --- | --- | --- | --- | --- | --- | --- |
| *qFML1-1* | - | Chr1_38408079 | 7.29 | 9.29 | - | - | 0.01 | - |
| *qFML2-1* | - | Chr2_4886951 | 6.82 | 8.14 | - | - | 0.01 | - |
| *qFML2-2* | - | Chr2_6284082 | 6.91 | 7.18 | - | - | 0.03 | - |
| *qFML3-1* | - | Chr3_29644652 | 6.87 | 12.13 | - | - | 0.07 | - |
| *qFML3-2* | LOC_Os03g53270 | Chr3_30563239 | 8.54 | 12.7 | - | - | 0.09 | Stem-specific protein TSJT1, putative, expressed |
|  | LOC_Os03g53340 | Chr3_30604849 | 8.57 | 14.87 | - | - | 0.08 | HSF-type DNA-binding domain containing protein, expressed |
| *qFML4-1* | LOC_Os04g16350 | Chr4_8888851 | 6.85 | 8.41 | C/T | G/D | 0.06 | Retrotransposon protein, putative, unclassified, expressed |
| *qFML4-2* | LOC_Os04g18680 | Chr4_10340699 | 6.99 | 9.29 | - | - | 0.03 | Retrotransposon protein, putative, unclassified, expressed |
| *qFML4-3* | LOC_Os04g19080 | Chr4_10588946 | 7.38 | 9.8 | A/T | I/F | 0.05 | Retrotransposon protein, putative, Ty3-gypsy subclass, expressed |
|  | - | Chr4_10692082 | 7.18 | 10.22 | - | - | 0.04 | - |
|  | LOC_Os04g19260 | Chr4_10705195 | 6.95 | 9.65 | - | - | 0.03 | Retrotransposon protein, putative, unclassified, expressed |
| *qFML6-1* | - | Chr6_26883178 | 7.72 | 8.49 | - | - | 0.05 | - |
| *qFML7-1* | LOC_Os07g18120 | Chr7_10729256 | 8.56 | 12.12 | - | - | 0.26 | Aldehyde oxidase, putative, expressed |
| *qFML7-2* | - | Chr7_10918516 | 7.93 | 9.62 | - | - | 0.4 | - |
| *qFML7-3* | - | Chr7_11242353 | 7.51 | 10.26 | - | - | 0.36 | - |
| *qFML7-4* | LOC_Os07g20590 | Chr7_11902494 | 8.91 | 11.05 | - | - | 0.37 | Retrotransposon, putative, centromere-specific |
| *qFML7-5* | - | Chr7_12704004 | 7.46 | 10.14 | - | - | 0.36 | - |
| *qFML7-6* | - | Chr7_13112154 | 8 | 13.96 | - | - | 0.07 | - |
|  | - | Chr7_13112337 | 7.84 | 12 | - | - | 0.1 | - |
|  | LOC_Os07g23270 | Chr7_13117230 | 9.44 | 12.49 | - | - | 0.09 | Transposon protein, putative, CACTA, En/Spm sub-class, expressed |
| *qFML7-7* | LOC_Os07g23740 | Chr7_13398939 | 7.12 | 9.39 | - | - | 0.38 | Sterol 3-beta-glucosyltransferase, putative, expressed |
|  | - | Chr7_13485801 | 7.1 | 7.25 | - | - | 0.06 | - |
|  | LOC_Os07g23944 | Chr7_13542834 | 7.06 | 9.63 | - | - | 0.38 | Glycosyl hydrolase, family 31, putative, expressed |
|  | - | Chr7_13554242 | 6.89 | 8.08 | - | - | 0.39 | - |
|  | - | Chr7_13582697 | 8.1 | 10.92 | - | - | 0.36 | - |
|  | LOC_Os07g23990 | Chr7_13599656 | 7.87 | 10.66 | - | - | 0.36 | Tetratricopeptide repeat domain containing protein, putative, expressed |
|  |  | Chr7_13602658 | 7.03 | 9.71 | A/T | M/L | 0.37 |  |
|  | - | Chr7_13618281 | 7.03 | 11.12 | - | - | 0.37 | - |
|  | - | Chr7_13619679 | 7.16 | 10.42 | - | - | 0.4 | - |
|  | - | Chr7_13626131 | 6.85 | 10.09 | - | - | 0.41 | - |
|  | - | Chr7_13626650 | 7.88 | 11.16 | - | - | 0.41 | - |
|  | - | Chr7_13630487 | 8.76 | 12.51 | - | - | 0.41 | - |
|  | - | Chr7_13636548 | 7.88 | 11.62 | - | - | 0.39 | - |
|  | LOC_Os07g24050 | Chr7_13638932 | 8.77 | 12.28 | - | - | 0.4 | Carboxyl-terminal proteinase, putative, expressed |
|  |  | Chr7_13640773 | 8.25 | 11.68 | - | - | 0.4 | - |
|  | - | Chr7_13657598 | 6.9 | 9.82 | - | - | 0.4 | - |
|  | - | Chr7_13658029 | 7.36 | 10 | - | - | 0.38 | - |
|  | - | Chr7_13681309 | 7.97 | 9.91 | - | - | 0.45 | - |
|  | - | Chr7_13681860 | 7.42 | 9.71 | - | - | 0.48 | - |
|  | - | Chr7_13682170 | 8.45 | 10.05 | - | - | 0.45 | - |
|  | - | Chr7_13689227 | 8.02 | 9.67 | - | - | 0.44 | - |
|  | - | Chr7_13690403 | 7.19 | 9.44 | - | - | 0.45 | - |
|  | - | Chr7_13691885 | 7.97 | 10.29 | - | - | 0.45 | - |
|  | - | Chr7_13693271 | 8.14 | 9.88 | - | - | 0.45 | - |
|  | LOC_Os07g24130 | Chr7_13694013 | 8.08 | 10.09 | - | - | 0.45 | Retrotransposon protein, putative, unclassified, expressed |
|  |  | Chr7_13695247 | 8.14 | 9.92 | - | - | 0.45 |  |
|  | - | Chr7_13697021 | 8.92 | 10.34 | - | - | 0.43 | - |
|  | - | Chr7_13697116 | 7.06 | 8.82 | - | - | 0.44 | - |
|  | LOC_Os07g24140 | Chr7_13698057 | 8.08 | 10.1 | - | - | 0.45 | Hypothetical protein |
|  |  | Chr7_13699059 | 8.02 | 9.81 | - | - | 0.45 |  |
|  |  | Chr7_13699476 | 7.81 | 9.87 | - | - | 0.45 |  |
|  |  | Chr7_13699956 | 7.03 | 8.78 | - | - | 0.45 |  |
|  | - | Chr7_13702499 | 7.7 | 9.28 | - | - | 0.44 | - |
|  |  | Chr7_13710068 | 7.24 | 9.35 | - | - | 0.44 | - |
|  | - | Chr7_13710109 | 8.89 | 10.37 | - | - | 0.44 | - |
|  | - | Chr7_13710231 | 8.22 | 9.98 | - | - | 0.44 | - |
|  | - | Chr7_13719118 | 6.93 | 7.63 | - | - | 0.25 | - |
|  | - | Chr7_13739871 | 7.17 | 9.62 | - | - | 0.39 | - |
|  | - | Chr7_13759824 | 6.96 | 9.59 | - | - | 0.38 | - |
|  | - | Chr7_13775181 | 7.16 | 9.79 | - | - | 0.38 | - |
|  |  | Chr7_13859018 | 6.94 | 6.27 | - | - | 0.49 | - |
| *qFML7-8* | LOC_Os07g25360 | Chr7_14494523 | 7.29 | 6.72 | - | - | 0.49 | Retrotransposon protein, putative, unclassified, expressed |
|  |  | Chr7_14497277 | 7.51 | 6.84 | - | - | 0.48 |  |
|  |  | Chr7_14498622 | 6.83 | 5.6 | C/A | Q/K | 0.47 |  |
|  | - | Chr7_14570298 | 6.88 | 9.9 | - | - | 0.39 | - |
| *qFML7-9* | - | Chr7_16048173 | 7.58 | 13.83 | - | - | 0.25 | - |
|  | - | Chr7_16064827 | 6.86 | 10.75 | - | - | 0.41 | - |
|  | LOC_Os07g27510 | Chr7_16074596 | 7.21 | 11.66 | - | - | 0.42 | Retrotransposon protein, putative, Ty1-copia subclass, expressed |
|  | LOC_Os07g27520 | Chr7_16083202 | 7.24 | 12.73 | - | - | 0.21 | Retrotransposon protein, putative, unclassified, expressed |
|  |  | Chr7_16083565 | 6.82 | 12.23 | - | - | 0.19 |  |
|  |  | Chr7_16084523 | 6.97 | 12.3 | - | - | 0.21 |  |
|  |  | Chr7_16084565 | 7.95 | 13.93 | - | - | 0.22 |  |
|  |  | Chr7_16085077 | 6.99 | 12.24 | C/T | Q/* | 0.22 |  |
|  |  | Chr7_16087845 | 7.28 | 12.94 | G/A | V/I | 0.22 |  |
|  |  | Chr7_16088389 | 7.45 | 12.75 | C/G | T/R | 0.23 |  |
|  | LOC_Os07g27530 | Chr7_16090802 | 7.2 | 13.01 | - | - | 0.22 | Retrotransposon protein, putative, unclassified, expressed |
|  |  | Chr7_16091498 | 6.93 | 12.26 | - | - | 0.22 |  |
|  | - | Chr7_16104317 | 6.85 | 12.24 | - | - | 0.25 | - |
|  | - | Chr7_16104569 | 7.11 | 12.37 | - | - | 0.23 | - |
|  | LOC_Os07g27570 | Chr7_16104688 | 7.2 | 13.14 | C/A | D/Y | 0.22 | Retrotransposon protein, putative, unclassified, expressed |
|  |  | Chr7_16105737 | 6.94 | 12.32 | - | - | 0.22 |  |
|  | - | Chr7_16121318 | 7.95 | 13.14 | - | - | 0.43 | - |
|  | - | Chr7_16130244 | 6.94 | 12.26 | - | - | 0.44 | - |
|  | - | Chr7_16133884 | 6.86 | 12.96 | - | - | 0.18 | - |
|  | LOC_Os07g27630 | Chr7_16136034 | 7.3 | 12.63 | - | - | 0.44 | Expressed protein |
|  | - | Chr7_16140582 | 7.83 | 13.05 | - | - | 0.44 | - |
|  | - | Chr7_16141848 | 7.02 | 12.37 | - | - | 0.43 | - |
|  | LOC_Os07g27720 | Chr7_16168840 | 7.07 | 12.59 | - | - | 0.45 | Retrotransposon protein, putative, Ty3-gypsy subclass, expressed |
|  | - | Chr7_16190387 | 6.85 | 12.04 | - | - | 0.43 | - |
| *qFML7-10* | - | Chr7_16389562 | 6.98 | 13.1 | - | - | 0.26 | - |
| *qFML12-1* | LOC_Os12g32430 | Chr12_19560950 | 8.14 | 10.23 | - | - | 0.02 | Retrotransposon protein, putative, unclassified, expressed |

^a^, -log(*p*) are association signals of CMLM using PC and kinship derived from group III.

^b^, -log(*p*) are association signals of GLM using PC derived from group III.
