# Supplementary material for: Genetic Architecture and Candidate Genes for Deep-Sowing Tolerance in Rice Revealed by Non-syn GWAS
Source: Front Plant Sci. 2018 Mar 16;9:332. doi: 10.3389/fpls.2018.00332 (PMC5864933; doi:10.3389/fpls.2018.00332)
Supplement: Supplementary file 8 [file Table8.DOCX]

**Table S8. Summary of SNPs associated with mesocotyl length by GWAS using CMLM and group** **I in *indica*.**

| QTL | Gene | Position | -log(*p*)^a^ | -log(*p*)^b^ | -log(*p*)^c^ | x^2^ value^d^ | SNP variation | Amino acid variation | MAF | Functional annotation |
| --- | --- | --- | --- | --- | --- | --- | --- | --- | --- | --- |
| *qIML7-1* | LOC_Os07g22360 | Chr7_12552125 | 5.88 | 6.84 | 5.1 | 9.26 | G/A | V/I | 0.07 | Expressed protein |
| *qIML7-2* | LOC_Os07g23990 | Chr7_13602658 | 6.61 | 8.43 | 6.53 | 26.52 | A/T | M/L | 0.45 | Tetratricopeptide repeat domain containing protein,  putative, expressed |
|  | LOC_Os07g24010 | Chr7_13611166 | 7.68 | 10.25 | 6.83 | 35.62 | C/T | R/Q | 0.39 | Hypothetical protein |
|  |  | Chr7_13611491 | 7.87 | 10.16 | 7.02 | 31.00 | A/T | S/T | 0.33 |  |
|  | LOC_Os07g24170 | Chr7_13728692 | 6.28 | 8.33 | 6.13 | 28.82 | T/A | N/K | 0.42 | Expressed protein |
|  |  | Chr7_13728704 | 5.9 | 7.91 | 5.73 | 26.45 | G/T | Q/H | 0.42 |  |
|  |  | Chr7_13729329 | 6.37 | 8.43 | 5.97 | 31.25 | G/A | V/M | 0.42 |  |
|  | LOC_Os07g24190 | Chr7_13746039 | 6.06 | 8.14 | 5.92 | 24.22 | C/T | M/I | 0.42 | CESA3 - cellulose synthase, expressed |
| *qIML7-3* | LOC_Os07g27610 | Chr7_16129890 | 6.45 | 8.33 | 5.22 | 33.63 | G/A | R/Q | 0.34 | Expressed protein |
|  | LOC_Os07g27630 | Chr7_16135146 | 5.91 | 7.35 | 4.58 | 29.53 | G/A | S/L | 0.29 | Expressed protein |
|  | LOC_Os07g27670 | Chr7_16148708 | 5.92 | 7.81 | 5.04 | 35.60 | C/T | P/L | 0.37 | WRKY115, expressed |

^a^, -log(*p*) are association signals of CMLM using PC and kinship derived from non-synonymous SNPs (group I).

^b^, -log(*p*) are association signals of GLM using PC derived from group I.

^c^, -log(*p*) are association signals of CMLM using PC and kinship derived from group III.

^d^, x^2^ value is the chi-squared test between polar pools in *indica*.
