# Supplementary material for: Genetic Architecture and Candidate Genes for Deep-Sowing Tolerance in Rice Revealed by Non-syn GWAS
Source: Front Plant Sci. 2018 Mar 16;9:332. doi: 10.3389/fpls.2018.00332 (PMC5864933; doi:10.3389/fpls.2018.00332)
Supplement: Supplementary file 9 [file Table9.DOCX]

**Table S9. Summary of SNPs associated with mesocotyl length by GWAS using CMLM and group II in *indica*.**

| QTL | Gene | Position | -log(*p*)^a^ | -log(*p*)^b^ | -log(*p*)^c^ | SNP variation | Amino acid variation | MAF | Functional annotation |
| --- | --- | --- | --- | --- | --- | --- | --- | --- | --- |
| *qIML1-1* | LOC_Os01g73530 | Chr1_42600590 | 6.57 | 6.71 | 5.96 | - | - | 0.01 | ABC transporter, ATP-binding protein, putative, expressed |
| *qIML3-1* | LOC_Os03g63090 | Chr3_35669651 | 6.48 | 6.09 | 5.86 | - | - | 0.06 | Actin-related protein 2/3 complex subunit 4, putative, expressed |
| *qIML7-1* | LOC_Os07g23740 | Chr7_13398939 | 7.25 | 9.33 | 6.52 | - | - | 0.44 | Sterol 3-beta-glucosyltransferase, putative, expressed |
|  | LOC_Os07g23960 | Chr7_13555256 | 6.82 | 8.87 | 6.35 | - | - | 0.37 | Transferase family protein, putative, expressed |
|  | LOC_Os07g23990 | Chr7_13602658 | 7.02 | 8.62 | 6.53 | A/T | M/L | 0.45 | Tetratricopeptide repeat domain containing protein, putative, expressed |
|  | LOC_Os07g24000 | Chr7_13607033 | 7.15 | 9.09 | 6.47 | - | - | 0.45 | AWPM-19-like membrane family protein, putative, expressed |
|  | LOC_Os07g24010 | Chr7_13611166 | 7.53 | 10.21 | 6.83 | C/T | R/Q | 0.39 | Hypothetical protein |
|  |  | Chr7_13611491 | 7.84 | 10.21 | 7.02 | A/T | S/T | 0.33 |  |
|  |  | Chr7_13612404 | 6.91 | 9.6 | 6.44 | - | - | 0.38 |  |
|  | LOC_Os07g24030 | Chr7_13624700 | 7.88 | 10.51 | 7.43 | - | - | 0.41 | Hypothetical protein |
|  | LOC_Os07g24050 | Chr7_13637538 | 7.22 | 9.91 | 6.7 | - | - | 0.39 | Carboxyl-terminal proteinase, putative, expressed |
|  |  | Chr7_13637833 | 6.69 | 9.3 | 6.17 | - | - | 0.39 |  |
|  | LOC_Os07g24140 | Chr7_13699658 | 6.57 | 7.99 | 5.82 | - | - | 0.4 | Hypothetical protein |
|  | LOC_Os07g24150 | Chr7_13706973 | 6.92 | 8.33 | 6.15 | - | - | 0.39 | Expressed protein |
|  | LOC_Os07g24170 | Chr7_13728692 | 6.55 | 8.42 | 6.13 | T/A | N/K | 0.42 | Expressed protein |
|  |  | Chr7_13729329 | 6.66 | 8.5 | 5.97 | G/A | V/M | 0.42 |  |
| *qIML7-2* | LOC_Os07g25440 | Chr7_14542597 | 6.89 | 8.87 | 6.29 | - | - | 0.42 | WD domain, G-beta repeat domain containing protein, expressed |
|  | LOC_Os07g25480 | Chr7_14593993 | 6.86 | 8.34 | 6.22 | - | - | 0.42 | Expressed protein |
|  |  | Chr7_14594035 | 7.29 | 8.99 | 6.58 | - | - | 0.42 |  |
|  | LOC_Os07g25484 | Chr7_14601372 | 6.78 | 8.28 | 6.23 | - | - | 0.42 | Expressed protein |
| *qIML11-1* | LOC_Os11g37880 | Chr11_22451344 | 6.97 | 6.2 | 6.28 | - | - | 0.02 | Stripe rust resistance protein Yr10, putative, expressed |

^a^,-log(*p*) are association signals of CMLM using PC and kinship derived from group II.

^b^, -log(*p*) are association signals of GLM using PC derived from group II.

^c^, -log(*p*) are association signals of CMLM using PC and kinship derived from group III.
