# Supplementary material for: Genetic Architecture and Candidate Genes for Deep-Sowing Tolerance in Rice Revealed by Non-syn GWAS
Source: Front Plant Sci. 2018 Mar 16;9:332. doi: 10.3389/fpls.2018.00332 (PMC5864933; doi:10.3389/fpls.2018.00332)
Supplement: Supplementary file 10 [file Table10.DOCX]

**Table S10. Summary of SNPs associated with mesocotyl length by GWAS using CMLM and group III in *indica*.**

| QTL | Gene | Position | -log(*p*)^a^ | -log(*p*)^b^ | SNP variation | Amino acid variation | MAF | Functional annotation |
| --- | --- | --- | --- | --- | --- | --- | --- | --- |
| *qIML5-1* | LOC_Os05g03350 | Chr5_1376864 | 6.6 | 8.94 | G/A | R/K | 0.02 | Retrotransposon protein, putative, unclassified, expressed |
| *qIML7-1* | - | Chr7_13549889 | 6.76 | 9.33 |  |  | 0.4 | - |
|  | - | Chr7_13565228 | 6.81 | 9.3 |  |  | 0.33 | - |
|  | - | Chr7_13582782 | 6.68 | 9.36 |  |  | 0.45 | - |
|  | LOC_Os07g23990 | Chr7_13604098 | 6.88 | 9.48 |  |  | 0.39 | Tetratricopeptide repeat domain containing protein, putative, expressed |
|  | - | Chr7_13604911 | 6.59 | 9.02 |  |  | 0.41 | - |
|  | - | Chr7_13606400 | 7.01 | 9.49 |  |  | 0.38 | - |
|  | - | Chr7_13610272 | 6.89 | 10.14 |  |  | 0.46 | - |
|  | LOC_Os07g24010 | Chr7_13611166 | 6.83 | 9.97 | C/T | R/Q | 0.39 | Hypothetical protein |
|  |  | Chr7_13611491 | 7.02 | 9.77 | A/T | S/T | 0.43 |  |
|  | LOC_Os07g24020 | Chr7_13612760 | 6.58 | 9.53 |  |  | 0.44 | Transposon protein, putative, CACTA, En/Spm sub-class, expressed |
|  |  | Chr7_13614871 | 6.66 | 9.71 | T/A | R/S | 0.46 |  |
|  |  | Chr7_13616751 | 6.94 | 9.96 |  |  | 0.39 |  |
|  | - | Chr7_13621224 | 6.94 | 9.91 |  |  | 0.27 | - |
|  | LOC_Os07g24050 | Chr7_13640129 | 7.24 | 10.09 |  |  | 0.45 | Carboxyl-terminal proteinase, putative, expressed |
| *qIML7-2* | - | Chr7_14582367 | 6.57 | 8.53 |  |  | 0.39 | - |
| *qIML12-1* | - | Chr12_1264441 | 7.28 | 6.28 |  |  | 0.42 | - |

^a^, -log(*p*) are association signals of CMLM using PC and kinship derived from group III.

^b^, -log(*p*) are association signals of GLM using PC derived from group III.
