# Supplementary material for: Genetic Architecture and Candidate Genes for Deep-Sowing Tolerance in Rice Revealed by Non-syn GWAS
Source: Front Plant Sci. 2018 Mar 16;9:332. doi: 10.3389/fpls.2018.00332 (PMC5864933; doi:10.3389/fpls.2018.00332)
Supplement: Supplementary file 11 [file Table11.DOCX]

**Table S11. Four known loci overlapping with *qML3-2* in previous bi-parental mapping.**

| Locus | Chromosome | Condition | Marker interval | LOD | PVE | Flank | Cross | Reference |
| --- | --- | --- | --- | --- | --- | --- | --- | --- |
| *QTL 3-3* | 3 | Water | BCD450-RZ393 | 3.04 | - | 30,936,578-35,827,028 | W1944/Pei-Kuh | Cai & Morishima, 2002 |
| *qML 3* | 3 | Water | RM426-RM448 | 8.93 | 24.97 | 27,588,613-32,250,282 | SN265/LTH | Huang *et al*., 2010 |
|  |  | Gibberellin | RM426-RM514 | 15.52 | 33.36 | 27,588,613-35,281,232 |  |  |
| *qMel-3* | 3 | Soil | RM3513-RM1238 | 4.2 | 11.5 | 25,112,673-32,077,117 | Nipponbare*/Kasalath* | Lee *et al*., 2012 |
|  | 3 | Agar |  | 7.5 | 20.8 |  |  |  |
| *qml 3-2* | 3 | FeSO4 | RZ328-RZ575 | 3.45 | 6 | 29,521,221-30,415,965 | Zhensan97B*/Miyang46* | Ouyang *et al*., 2005 |

* Sequenced in the “3K Rice Genome Project”.
