# Supplementary material for: Genetic Architecture and Candidate Genes for Deep-Sowing Tolerance in Rice Revealed by Non-syn GWAS
Source: Front Plant Sci. 2018 Mar 16;9:332. doi: 10.3389/fpls.2018.00332 (PMC5864933; doi:10.3389/fpls.2018.00332)
Supplement: Supplementary file 12 [file Table12.DOCX]

**Table S12. Independent-sample T-tests for mesocotyl length differences between alleles of 3 InDel in LOC_Os03g53340.**

| Position | | Chr3_30605444 | Chr3_30605446 | Chr3_30606411 |
| --- | --- | --- | --- | --- |
| Allele 1 | | TGTCGCC | TCGCCGCCGC | CGCCGCCGCCGCG |
| Allele 2 | | T | TCGC | C |
| *Ind.* | ML of Allele 1 (cm) | 1.51 (166) | 1.45 (152) | 1.51 (161) |
|  | ML of Allele 2 (cm) | 1.31 (16) | 1.71 (55) | 1.68 (65) |
|  | Sig.(2-tailed) | 0.42 | 0.1 | 0.29 |
| *Jap.* | ML of Allele 1 (cm) | 1.41 (142) | 1.42 (137) | 1.43 (143) |
|  | ML of Allele 2 (cm) | - | - | - |
|  | Sig.(2-tailed) | - | - | - |

Data in parentheses show number of accessions.
