# Supplementary material for: Genetic Architecture and Candidate Genes for Deep-Sowing Tolerance in Rice Revealed by Non-syn GWAS
Source: Front Plant Sci. 2018 Mar 16;9:332. doi: 10.3389/fpls.2018.00332 (PMC5864933; doi:10.3389/fpls.2018.00332)
Supplement: Supplementary file 13 [file Table13.DOCX]

**Table S13. Genotypes of 7 non synonymous SNPs above threshold among four accessions in two previous bi-parental mappings.**

| Accession | Chr7_13602658 | Chr7_13611491 | Chr7_13728692 | Chr7_13728704 | Chr7_13729329 | Chr7_13746039 | Chr7_14579544 | Reference |
| --- | --- | --- | --- | --- | --- | --- | --- | --- |
| Kasalath | AT | AT | AT | GT | AG | CT | AG | Lee *et al*., 2012 |
| Nipponbare | AA | AA | TT | GG | GG | CC | GG |  |
| Zhensan97B | TT | AA | AA | TT | AA | TT | AA | Ouyang *et al*., 2005 |
| Miyang46 | TT | AA | AA | TT | AA | TT | AA |  |
