# Supplementary material for: Genetic Architecture and Candidate Genes for Deep-Sowing Tolerance in Rice Revealed by Non-syn GWAS
Source: Front Plant Sci. 2018 Mar 16;9:332. doi: 10.3389/fpls.2018.00332 (PMC5864933; doi:10.3389/fpls.2018.00332)
Supplement: Supplementary file 14 [file Table14.DOCX]

**Table S14. Summary of 12 non-synonymous SNPs in *OsML2*.**

| Position | full population | | | *Indica* | | | MAF | Amino acid variation | SNP variation |
| --- | --- | --- | --- | --- | --- | --- | --- | --- | --- |
|  | -log(*p*)^h^ | -log(*p*)^i^ | -log(*p*)^j^ | -log(*p*)^h^ | -log(*p*)^i^ | -log(*p*)^j^ |  |  |  |
| Chr7_13611166 | 5.69 | 5.78 | 4.68 | 7.68 | 7.53 | 6.83 | 0.45 | R/Q | C/T |
| Chr7_13611223 | 4.29 | 4.39 | 4.08 | 2.34 | 2.47 | 2.32 | 0.14 | R/H | C/T |
| Chr7_13611283 | 0.58 | 0.58 | 0.67 | 0.19 | 0.1 | 0.28 | 0.35 | T/I | G/A |
| Chr7_13611284 | 0.72 | 0.74 | 0.82 | 0.11 | 0.01 | 0.12 | 0.35 | T/P | T/G |
| Chr7_13611304 | 4.91 | 5.38 | 5.09 | 4.41 | 4.41 | 3.85 | 0.16 | I/T | A/G |
| Chr7_13611452 | 1.22 | 1.29 | 1.32 | 0.05 | 0.14 | 0.03 | 0.34 | D/N | C/T |
| Chr7_13611463 | 4.82 | 5 | 4.61 | 2.68 | 2.67 | 2.42 | 0.15 | Q/R | T/C |
| Chr7_13611487 | 0.94 | 0.97 | 1.03 | 0.05 | 0.02 | 0.05 | 0.34 | L/P | A/G |
| Chr7_13611491 | 10.51 | 11.2 | 10.35 | 7.87 | 7.84 | 7.02 | 0.26 | S/T | A/T |
| Chr7_13611508 | 0.88 | 0.91 | 0.98 | 0.02 | 0.05 | 0.03 | 0.34 | G/E | C/T |
| Chr7_13611535 | 5.26 | 5.82 | 5.45 | 4.55 | 4.62 | 4.1 | 0.16 | S/L | G/A |
| Chr7_13611536 | 0.72 | 0.73 | 0.8 | 0.22 | 0.15 | 0.23 | 0.34 | S/P | A/G |

^h^, -log(*p*) are association signals of CMLM using PC and kinship derived from group I.

^i^, -log(*p*) are association signals of CMLM using PC derived from group II.

^j^, -log(*p*) are association signals of CMLM using PC and kinship derived from group III.
