# Supplementary material for: Genetic Architecture and Candidate Genes for Deep-Sowing Tolerance in Rice Revealed by Non-syn GWAS
Source: Front Plant Sci. 2018 Mar 16;9:332. doi: 10.3389/fpls.2018.00332 (PMC5864933; doi:10.3389/fpls.2018.00332)
Supplement: Supplementary file 15 [file Image1.PDF]

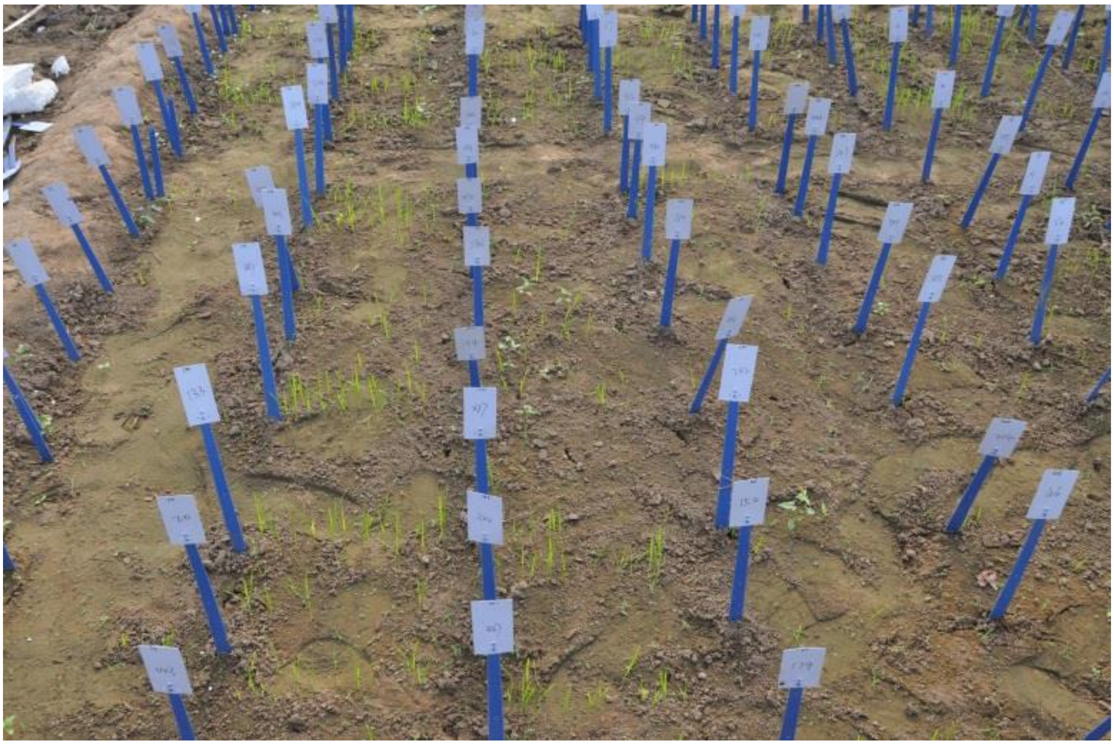

Figure S1. View of emergence differences in field experiment with a 10 cm soil cover for mesocotyl length phenotyping of 621 cultivated rice accessions. Ten seeds were sown in each row.
