# Supplementary figures and images for: Genetic Architecture and Candidate Genes for Deep-Sowing Tolerance in Rice Revealed by Non-syn GWAS
Source: Front Plant Sci. 2018 Mar 16;9:332. doi: 10.3389/fpls.2018.00332 (PMC5864933; doi:10.3389/fpls.2018.00332)

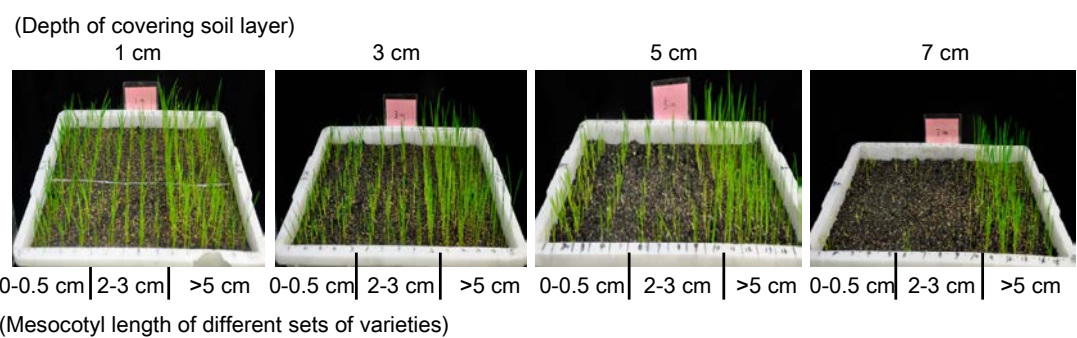

1  
2  
3  
4

**Figure S2. Soil culture experiments in plastic boxes with different depths of soil cover.**

Supplement: Supplementary file 16 [file Image2.PDF]
