# Supplementary material for: Genetic Architecture and Candidate Genes for Deep-Sowing Tolerance in Rice Revealed by Non-syn GWAS
Source: Front Plant Sci. 2018 Mar 16;9:332. doi: 10.3389/fpls.2018.00332 (PMC5864933; doi:10.3389/fpls.2018.00332)
Supplement: Supplementary file 17 [file Image3.PDF]

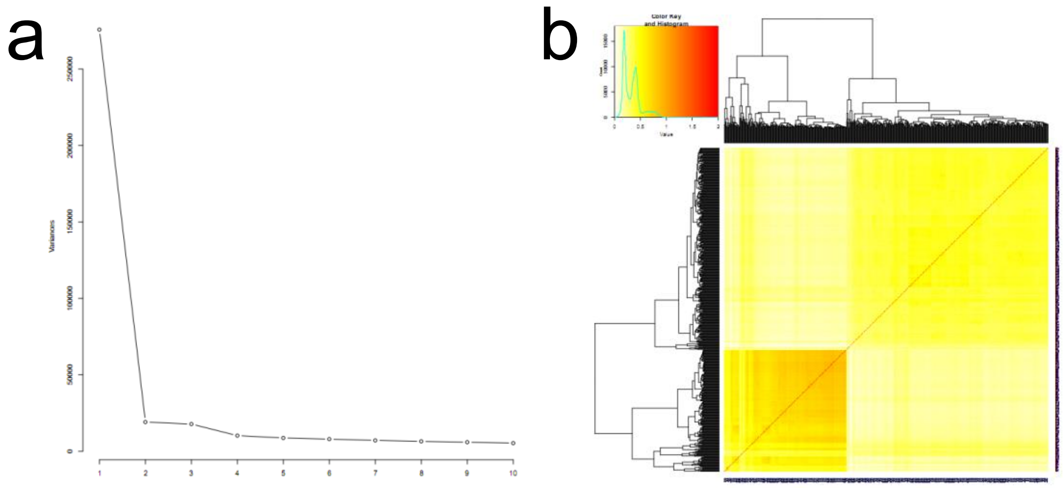

Figure S3. Principal component analysis and relative kinship analysis of the full population. (a) The genetic variation explained by each of the first 10 principal conponents (PC). **(b)** Kinship plot of 621 rice accessions.
