# Supplementary material for: Genetic Architecture and Candidate Genes for Deep-Sowing Tolerance in Rice Revealed by Non-syn GWAS
Source: Front Plant Sci. 2018 Mar 16;9:332. doi: 10.3389/fpls.2018.00332 (PMC5864933; doi:10.3389/fpls.2018.00332)
Supplement: Supplementary file 19 [file Image5.PDF]

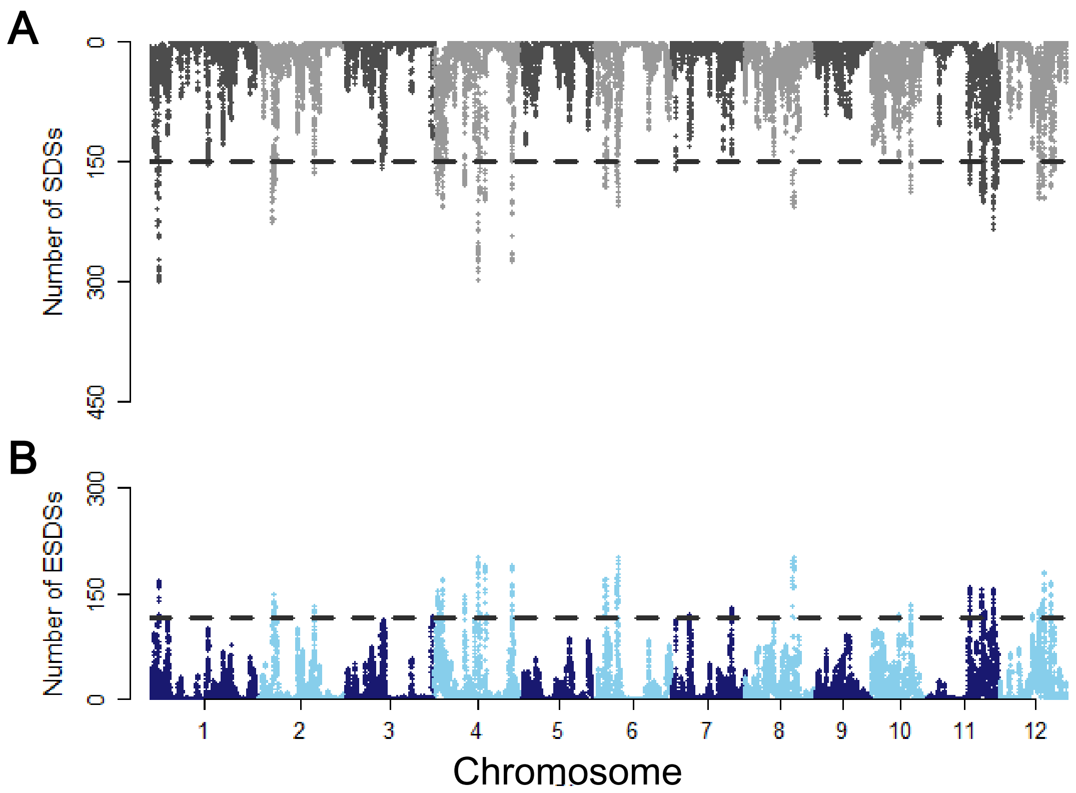


**Figure S5. SDS and ESDS distribution along the genomes for mesocotyl length in *japonica*.** Distributions of **(A)** SDSs and **(B)** ESDSs along the *japonica* genome. SDS and ESDS show SNPs with significant and highly significant differences ($p$ < 0.05; $p$ < 0.01) in allele frequency between polar pools. For each 500 kb sliding window, the numbers of SDSs and ESDSs were plotted on the entire genome. The sliding step is 50 kb. Adjacent chromosomes are delineated using different colors. Horizontal black lines show the thresholds for the 99$^{th}$ percentile of 10,000 permutations of the SDS and ESDS numbers.
