# Supplementary material for: Genetic Architecture and Candidate Genes for Deep-Sowing Tolerance in Rice Revealed by Non-syn GWAS
Source: Front Plant Sci. 2018 Mar 16;9:332. doi: 10.3389/fpls.2018.00332 (PMC5864933; doi:10.3389/fpls.2018.00332)
Supplement: Supplementary file 20 [file Image6.PDF]

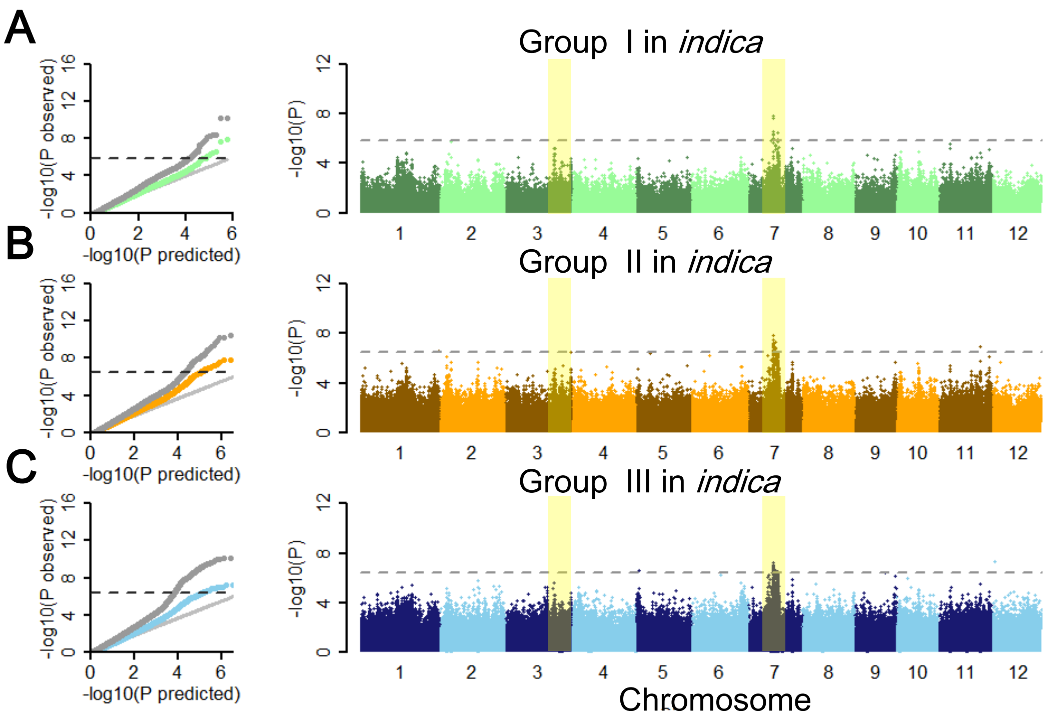

Figure S6. Genome-wide association studies of mesocotyl length under CMLM in *indica* using three sets of SNPs. Quantile-quantile plots and Manhattan plots of the CMLM using groups **(A)** I, **(B)** II and **(C)** III in *indica*. In quantile-quantile plots, gray dots show GLM, and other colored points show CMLM. The horizontal black dashed lines in Manhattan plots of CMLM show thresholds at $p = 0.01$ after Bonferroni-adjusted multiple test correction. Yellow stripes show two important signals in 6 GWAS using three sets of SNPs in full population and *indica*.
