# Supplementary material for: Genetic Architecture and Candidate Genes for Deep-Sowing Tolerance in Rice Revealed by Non-syn GWAS
Source: Front Plant Sci. 2018 Mar 16;9:332. doi: 10.3389/fpls.2018.00332 (PMC5864933; doi:10.3389/fpls.2018.00332)
Supplement: Supplementary file 22 [file Image8.PDF]

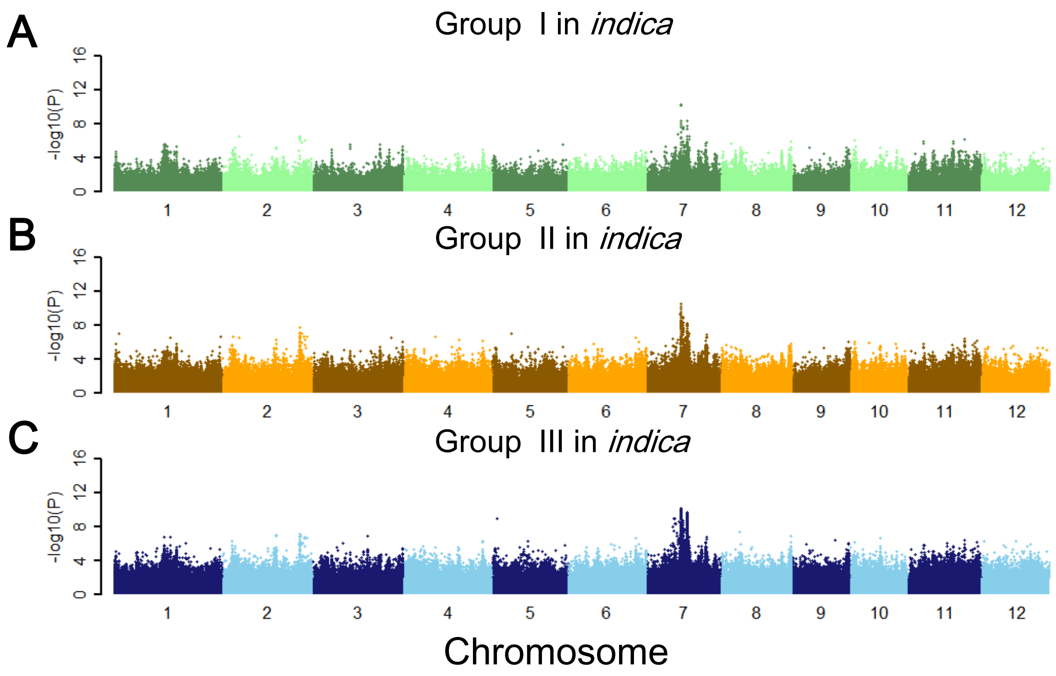

Figure S8. Genome-wide association studies of mesocotyl length under GLM in *indica* using three sets of SNPs. Manhattan plots for mesocotyl length using groups **(A)** I, **(B)** II and **(C)** III in *indica*.
