# Supplementary material for: Genetic Architecture and Candidate Genes for Deep-Sowing Tolerance in Rice Revealed by Non-syn GWAS
Source: Front Plant Sci. 2018 Mar 16;9:332. doi: 10.3389/fpls.2018.00332 (PMC5864933; doi:10.3389/fpls.2018.00332)
Supplement: Supplementary file 23 [file Image9.PDF]

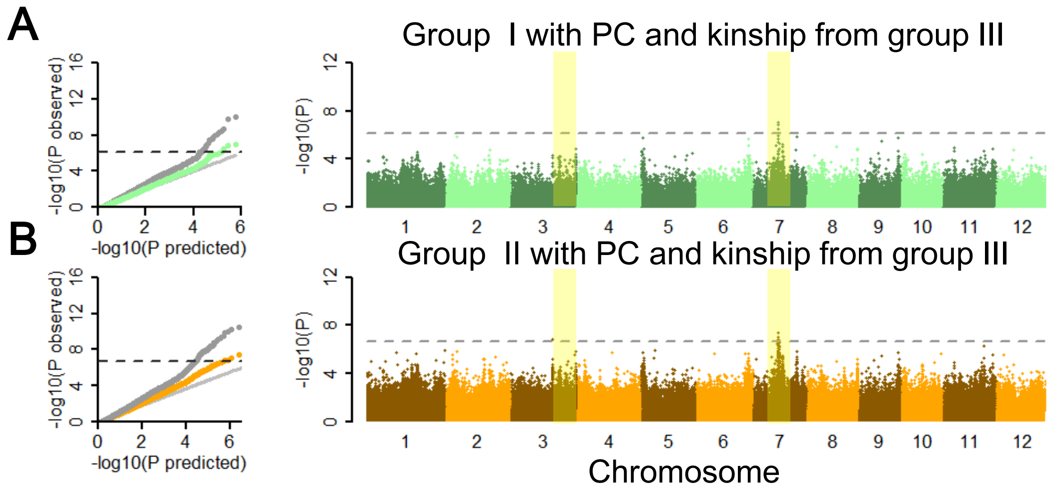

Figure S9. Genome-wide association studies of mesocotyl length under CMLM in *indica* using three sets of SNPs with PC and kinship derived from group III. Quantile-quantile plots and Manhattan plots of CMLM using groups **(A)** I and **(B)** II with PC and kinship derived from group III. In quantile-quantile plots, gray dots show GLM, and other colored points show CMLM. The horizontal black dashed lines in Manhattan plots of CMLM show thresholds at p = 0.01 after Bonferroni-adjusted multiple test correction. Yellow stripes show two important signals in 6 GWAS using three sets of SNPs in full population and *indica*.
