# Supplementary material for: Genetic Architecture and Candidate Genes for Deep-Sowing Tolerance in Rice Revealed by Non-syn GWAS
Source: Front Plant Sci. 2018 Mar 16;9:332. doi: 10.3389/fpls.2018.00332 (PMC5864933; doi:10.3389/fpls.2018.00332)
Supplement: Supplementary file 24 [file Image10.PDF]

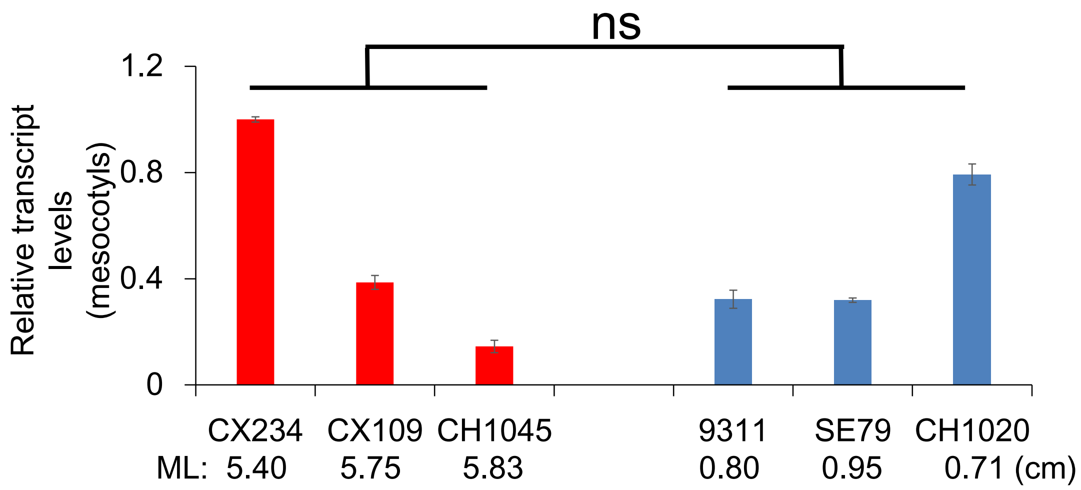

Figure S10. Expression analysis of LOC_Os03g53340 in mesocotyls from 6 accessions with long- and short-mesocotyls. Name and mesocotyl length of accessions are plotted on the *X*-axis. Red and blue bars show long- and short-mesocotyl accessions, respectively. Data represent means ± s.d (n = 3).
